# What predicts people’s belief in COVID-19 misinformation? A retrospective study using a nationwide online survey among adults residing in the United States

**DOI:** 10.1186/s12889-022-14431-y

**Published:** 2022-11-18

**Authors:** Sooyoung Kim, Ariadna Capasso, Shahmir H. Ali, Tyler Headley, Ralph J. DiClemente, Yesim Tozan

**Affiliations:** 1 Department of Public Health Policy and Management, New York, School of Global Public Health, New York University, 708 Broadway, 4th floor, New York, NY 10003 USA; 2 Department of Social and Behavioral Sciences, School of Global Public Health, New York University, New York, NY USA; 3 Global and Environmental Public Health Program, School of Global Public Health, New York University, New York, NY USA

**Keywords:** COVID-19, Misinformation, Infodemic, LASSO

## Abstract

**Background:**

Tackling infodemics with flooding misinformation is key to managing the COVID-19 pandemic. Yet only a few studies have attempted to understand the characteristics of the people who believe in misinformation.

**Methods:**

Data was used from an online survey that was administered in April 2020 to 6518 English-speaking adult participants in the United States. We created binary variables to represent four misinformation categories related to COVID-19: general COVID-19-related, vaccine/anti-vaccine, COVID-19 as an act of bioterrorism, and mode of transmission. Using binary logistic regression and the LASSO regularization, we then identified the important predictors of belief in each type of misinformation. Nested vector bootstrapping approach was used to estimate the standard error of the LASSO coefficients.

**Results:**

About 30% of our sample reported believing in at least one type of COVID-19-related misinformation. Belief in one type of misinformation was not strongly associated with belief in other types. We also identified 58 demographic and socioeconomic factors that predicted people’s susceptibility to at least one type of COVID-19 misinformation. Different groups, characterized by distinct sets of predictors, were susceptible to different types of misinformation. There were 25 predictors for general COVID-19 misinformation, 42 for COVID-19 vaccine, 36 for COVID-19 as an act of bioterrorism, and 27 for mode of COVID-transmission.

**Conclusion:**

Our findings confirm the existence of groups with unique characteristics that believe in different types of COVID-19 misinformation. Findings are readily applicable by policymakers to inform careful targeting of misinformation mitigation strategies.

**Supplementary Information:**

The online version contains supplementary material available at 10.1186/s12889-022-14431-y.

## Background

The COVID-19 infodemic, defined as “too much information including false or misleading information in digital and physical environments during a disease outbreak,” [1] has been one of the primary impediments to curbing the persisting COVID-19 pandemic by polarizing opinions and affecting compliance with public health measures [2]. Specifically, the proliferation of pandemic-related misinformation have led to the adoption of conspiracy theories and often negatively affected health-related decision-making [3, 4]. For example, a quasi-experimental study conducted in the United States (US) and the United Kingdom (UK) found that people who were exposed to misinformation had a lower intention of getting vaccinated against COVID-19; further, the proportion of vaccine-hesitant people had grown in tandem with the spread of misinformation [5]. Moreover, misinformation impacted sociodemographic groups differently [5]. In the same study, certain sociodemographic characteristics, such as race, employment status, and educational attainment, modified the association between exposure to misinformation and intention of getting vaccinated significantly, highlighting the importance of targeted communication and intervention to achieving herd immunity.

The problem of widespread misinformation is not new although it is particularly concerning in the United States during the current pandemic where the prevalence of COVID-19 misinformation is estimated to be one of the highest across all countries [6, 7]. With user-generated content inundating online platforms like social media, effectively countering misinformation has long been a challenge in the field of public health [8–11]. One demonstrated method to thwart misinformation is through active and strategic responses based on demonstrating misinformation’s falsehood [3, 4] and presenting the correct information through targeted dissemination [4, 12–14]. Understanding who believes in what type(s) of misinformation is therefore critical to creating, targeting, and executing a counter-misinformation strategy.

However, studies on COVID-19 misinformation have primarily focused on profiling the types and sources of misinformation [15–18], detecting misinformation using machine learning algorithms [19–23], or exploring the behavior-related consequences of misinformation [15, 24–28]. Only a few have attempted to understand the characteristics of the people or communities who believe in COVID-19 misinformation [29, 30]. Roozenbeek and colleagues used a cross-sectional survey from five countries (Ireland, the US, Mexico, Spain, and the UK) to identify the predictors of susceptibility to misinformation [29]. They regressed an average susceptibility score on a pre-selected set of predictors and found that susceptibility to misinformation was negatively associated with compliance with COVID-19 public health guidance, including willingness to get vaccinated. Lobato et al. conducted an exploratory canonical correlation analysis to identify individual characteristics associated with willingness to share misinformation [30]. The authors found that certain aspects of political beliefs predicted tendencies to disseminate misinformation.

This study expands on prior studies on COVID-19 misinformation in two important ways. First, we employed a novel model selection approach to widen the scope of potential predictors rather than narrowing the scope to a pre-selected subset. Second, we explored people’s belief in different types of misinformation, informed by the published literature [15, 17, 29, 31], rather than aggregating misinformation into a single index. The primary aim of the study was to provide insights into who believes in COVID-19 misinformation so as to inform the design and targeting of misinformation mitigation strategies.

## Methods

### Data

This study is a secondary analysis of data from an online survey conducted in April 2020. The primary aim of the survey was to collect and analyze data on COVID-19-related knowledge, beliefs, and behaviors among the US adult population during the early days of the pandemic [32]. In short, the questionnaire was developed based on the Health Belief Model [33] and included validated scales from the literature [32]. Participants were recruited using a convenience sampling approach via Facebook and its affiliated platforms, namely Instagram, Facebook Messenger, and Facebook Audience Network, through social media advertisement campaigns. In particular, the data used in this analysis is from the second wave of the survey that happened between April 16–21, 2020. In this wave, the questionnaire was completed by 6518 voluntary and eligible participants. Eligibility criteria included being an English-speaking adult (aged 18 years and older) who was physically residing in the US. While most of the survey design and administration methods are consistent with the first wave, which was conducted in March 2020 [32], a number of questions are excluded or newly added in the second wave. To illustrate the discrepancy, the complete survey questionnaire used in the second wave with all the changes highlighted is provided in the supplementary material (Additional file 1, Table S1–1). The analysis only included participants who expressed informed consent and provided a complete response to all the questions that were used to define the variables for the analysis. We checked the pattern of missingness in the data, specifically whether data are missing completely at random (MCAR) or missing at random (MAR) [34, 35]. The study protocol was reviewed and deemed exempt by New York University’s Institutional Review Board.

### Belief in misinformation

Belief in misinformation is defined as believing something specific that is false or inaccurate [1, 36]. We derived four variables representing people’s self-reported belief in different types of misinformation—general, bioterrorism, anti-vaccine, and transmission mode—from our survey. Constrained by the lack of theoretical ground that categorizes the different types of COVID-19 misinformation, we formed these four variables by reviewing the published literature on COVID-19 misinformation and taking as a basis the most commonly identified types of misinformation in these studies [15, 17, 29, 31]. First, we created a variable on belief in generalized misinformation by identifying respondents whose COVID-19 knowledge scores were in the bottom quartile [37] and who responded “yes” to the statement “I believe the information I get about Coronavirus is accurate.” Prior studies showed that COVID-19 misinformation clustered into distinct thematic categories and that different “dubious beliefs” about COVID-19 attracted distinct groups of people [15, 17, 31]. While there is no single agreed-upon approach to this categorization, the most common categories of misinformation include the modes of transmission; miracle cures or treatments; anti-vaccine; political conspiracy theories; racism; and bioterrorism [15–18, 29, 31]. Next, using the variables in our data set we created binary variables for three dubious beliefs - namely, belief in COVID-19 as bioterror, anti-vaccine misinformation, and transmission mode misinformation.

First, we classified participants as believing in misinformation on bioterrorism if they responded “strongly agree” or “agree” to the statement “I think that Coronavirus was released as an act of bioterrorism.” Second, we classified participants as believing in misinformation related to a COVID-19 vaccine if they responded “not likely” to the question “how likely would you be to get a Coronavirus vaccine if it was recommended by: doctor/medical provider?” We note that though no vaccine had been released at the time of this survey, there was already a significant volume of misinformation about possible COVID-19 vaccines, such as the conspiracy theory that the vaccines would include a geolocation-tracking microchip; thus, reticence to get a hypothetical vaccine that was hypothetically endorsed by respondents’ medical providers was categorized as belief in misinformation. To do so, we used the theory of rationality in health decision making and the health belief model that describe the cognitive pathway from perception to behavior change [38–40] and hypothesized that reticence for receiving a recommended vaccine will be formed when an individual’s perceived risk of the vaccine formed by misinformation exceeds its perceived benefit manifested by a medical provider’s recommendation. We further assumed that, during the survey period during which there was no notion of vaccine supply shortage, all individuals would have been willing to receive a recommended vaccine as long as the perceived benefit exceeded the perceived risk. In other words, we ruled out some altruistic scenarios observed later during the pandemic under the perceived vaccine supply shortage, where individuals had reservations in getting vaccinated to prioritize access to vulnerable individuals. Third, we classified participants as believing in misinformation on the mode of COVID-19 transmission if they: 1) answered “no” to “practicing social distancing” and “wearing a face mask or covering when they leave home,” and 2) responded “strongly disagree” or “disagree” to the statement “if I were ORDERED to quarantine myself due to Coronavirus, I would do so.”

### Potential predictors of belief in misinformation

We included 66 variables from the survey as potential predictors of people’s belief in misinformation. These variables depicted participants’ sociodemographic characteristics, including, but not limited to, age, sex, race/ethnicity, highest educational attainment, annual household income, marital status, residence area, political affiliation, COVID-19-related knowledge levels, information-seeking patterns, and beliefs and perceptions about the COVID-19 disease. A complete list of the variables, their definitions, and participants’ responses are summarized in the supplementary material (Additional file 2, Table S2).

### Data analysis

We used binary logistic regressions and the LASSO (Least Absolute Shrinkage and Selection Operator) regularization to select important predictors of belief in misinformation among the initial set of 66 variables (*p* = 66). Since COVID-19-related knowledge level, coded as as a score ranging between 0 and 21, was used to create one of the four outcome variables (i.e., general COVID-19 misinformation), we excluded this variable and used the remaining 65 variables (*p* = 65) when performing the analysis for this outcome. The equation below illustrates the logistic regression of the outcome variable of belief in misinformation (Y) using the set of *p* predictors (*X*_1_, *X*_2_, …, *X*_*p*_).

$$\log \left(\frac{\mathit{\Pr}\left(Y=1\right)}{1-\mathit{\Pr}\left(Y=1\right)}\right)={\beta}_0+{\beta}_1{X}_1+{\beta}_2{X}_2+\dots +{\beta}_p{X}_p$$

LASSO regularization reduces the high dimensionality of the data. It is a variable selection method that has been increasingly used in place of the traditional stepwise selection approach (i.e., backward selection, forward selection) [34]. It has been shown to improve the model fit by avoiding stepwise selection’s path-dependency and reducing overfitting issues by using a cross-validation approach [34]. In brief, the LASSO method enables the selection of a model with the best fitting subset of explanatory variables by introducing the penalty term $\lambda \sum_{j=1}^p\mid {\beta}_j\mid$ into the regression equation. The method estimates the regression coefficients (*β*_*j*_) by minimizing the sum of squared residuals ($\sum_{i=1}^n{\left({y}_i-\hat{y_i}\right)}^2$) while shrinking some of the coefficient estimates to zero when the tuning parameter *λ* is sufficiently large. As a result, the LASSO logistic regression yields a sparse model with only a subset of variables.

$$\sum_{i=1}^n{\left({y}_i-\hat{y_i}\ \right)}^2+\lambda \sum_{j=1}^p\left|{\beta}_j\right|$$

We used the glmnet package in R [41] to conduct the LASSO logistic regression. We used 10-fold cross-validation to identify the optimal value of *λ*. We then followed the vector bootstrapping approach proposed by Laurin et al. [42] to estimate the standard error (SE) and the 95% confidence interval (CI) of the LASSO coefficients ($\hat{\beta_j}$). We took this additional step because, first, we wanted to account for the low variable selection precision (VSP), the percent of true important predictors among the model-selected predictors, following the LASSO approach [43–45]. Second, we wanted to improve the interpretability of the model by constructing the 95% CI for the LASSO estimate. By doing so, the results can be interpreted similarly to the conventional frequentist framework [42]. Specifically, we used the nested cross-validated selection methods for *λ* (described as Method 3 in Laurin et al., 2016), which leads to larger SEs than the fixed *λ* bootstrapping (Method 2) [42]. Using Monte Carlo simulation, we estimated the coefficients and calculated the 95% CI of the coefficients based on an approximate inverted z-test ($\hat{\beta_j}\pm {z}_{\alpha /2}\ast SE\ast \Big(\hat{\beta_j\Big)}$), where *z*_*α*/2_ is the $\frac{\alpha }{2}$ quantile of a standard normal distribution and *α* = 0.05 for the 95% CI. We kept the variables that had non-zero coefficients with their 95% confidence interval not crossing the value zero. Thus, the final set of variables retained in the model further improve the VSP. The larger estimated SE from the nested cross-validation (CV) approach for *λ* also made the CI-based selection of variables more conservative than the fixed *λ* [42]

## Results

### Descriptive statistics

Table 1 summarizes the descriptive statistics of the survey participants. Of the 6518 survey participants, only 2793 (42.9%) provided a complete response to the variables used in the analysis. Upon checking the missingness of the data, while most explanatory variables were missing completely at random (MCAR), some variables, namely highest educational attainment, annual household income, employment status, and type of residence, showed a missingness pattern at random (MAR) (Additional file 2, Figs. S2–1 and S2–2). The distribution of several sociodemographic characteristics such as sex, race, highest educational attainment, and political affiliation to Democratic Party was similar between the overall sample and the regression sample (the data subset which included only complete responses). However, the distribution of age group, marital status, the number of children and people in a household, employment status, annual household income, political affiliation to Republican Party, and geographic region and type of residence were significantly different between two samples.

**Table 1:** Descriptive statistics of COVID-19 survey responses in April 2020 for the total sample (*N* = 6518) and the regression sample with complete data only (*N* = 2793)

	Total sample (***N*** = 6518)	Regression sample (***N*** = 2793)	***p***-value
**Sex**			**0.951**
Female	3717 (57.0%)	1610 (57.6%)	
Male	2738 (42.0%)	1183 (42.4%)	
Missing	63 (1.0%)		
**Age group**			**< 0.001**
18–29 years old	343 (5.3%)	120 (4.3%)	
30–39 years old	735 (11.3%)	372 (13.3%)	
40–49 years old	997 (15.3%)	495 (17.7%)	
50–59 years old	1814 (27.8%)	863 (30.9%)	
60–69 years old	1967 (30.2%)	755 (27.0%)	
70–79 years old	605 (9.3%)	179 (6.4%)	
80+ years old	57 (0.9%)	9 (0.3%)	
**Race**			**0.051**
White, Non-Hispanic	6012 (92.2%)	2634 (94.3%)	
Hispanic/Latinx	169 (2.6%)	52 (1.9%)	
Interracial, Mixed race, or Other	190 (2.9%)	63 (2.3%)	
Asian/Pacific Islander	50 (0.8%)	15 (0.5%)	
Black, Non-Hispanic	53(0.8%)	12 (0.4%)	
Native American or American Indian	44 (0.7%)	17 (0.6%)	
**Currently married**			**< 0.001**
No	1475 (22.6%)	492 (17.6%)	
Yes	3585 (55.0%)	2301 (82.4%)	
Missing	1458 (22.4%)		
**Children under 18 in the household**			**< 0.001**
No	4253 (65.3%)	1893 (67.8%)	
Yes	1477 (22.7%)	900 (32.2%)	
Missing	788 (12.1%)		
**Number of people in the household**			**0.015**
Mean (SD)	3.16 (1.70)	2.84 (1.26)	
**Employment status**			**< 0.001**
Employed	2845 (43.6%)	1832 (65.6%)	
Student/Unpaid work	280 (4.3%)	140 (5.0%)	
Not working/Unemployed	635 (9.7%)	325 (11.6%)	
Retired	1300 (19.9%)	496 (17.8%)	
Missing	1458 (22.4%)		
**Highest educational attainment**			**0.050**
High school degree / GED or less	516 (7.9%)	264 (9.5%)	
Some college / Associate’s degree	1720 (26.4%)	944 (33.8%)	
Bachelor’s degree or higher	2792 (42.8%)	1585 (56.7%)	
Missing	1490 (22.9%)		
**Annual household income**			**< 0.001**
Less than $30,000	580 (8.9%)	233 (8.3%)	
$30,000 to less than $50,000	671 (10.3%)	378 (13.5%)	
$50,000 to less than $75,000	767 (11.8%)	477 (17.1%)	
$75,000 to less than $100,000	900 (13.8%)	614 (22.0%)	
$100,000 or more	1419 (21.8%)	1091 (39.1%)	
Missing	2181 (33.5%)		
**Democrat (political affiliation)**			**0.675**
No	3103 (47.6%)	1716 (61.4%)	
Yes	1925 (29.5%)	1077 (38.6%)	
Missing	1490 (22.9%)		
**Republican (political affiliation)**			**< 0.001**
No	3806 (58.4%)	2043 (73.1%)	
Yes	1222 (18.7%)	750 (26.9%)	
Missing	1490 (22.9%)		
**Region of residence**			**0.042**
Northeast	1379 (21.2%)	772 (27.6%)	
Midwest	1308 (20.1%)	756 (27.1%)	
South	1379 (21.2%)	746 (26.7%)	
West	994 (15.3%)	519 (18.6%)	
Missing	1458 (22.4%)		
**Type of residence**			**0.009**
Suburban	2697 (41.4%)	1538 (55.1%)	
Urban	770 (11.8%)	395 (14.1%)	
Rural	1593 (24.4%)	860 (30.8%)	
Missing	1458 (22.4%)		

**Fig. 1:**
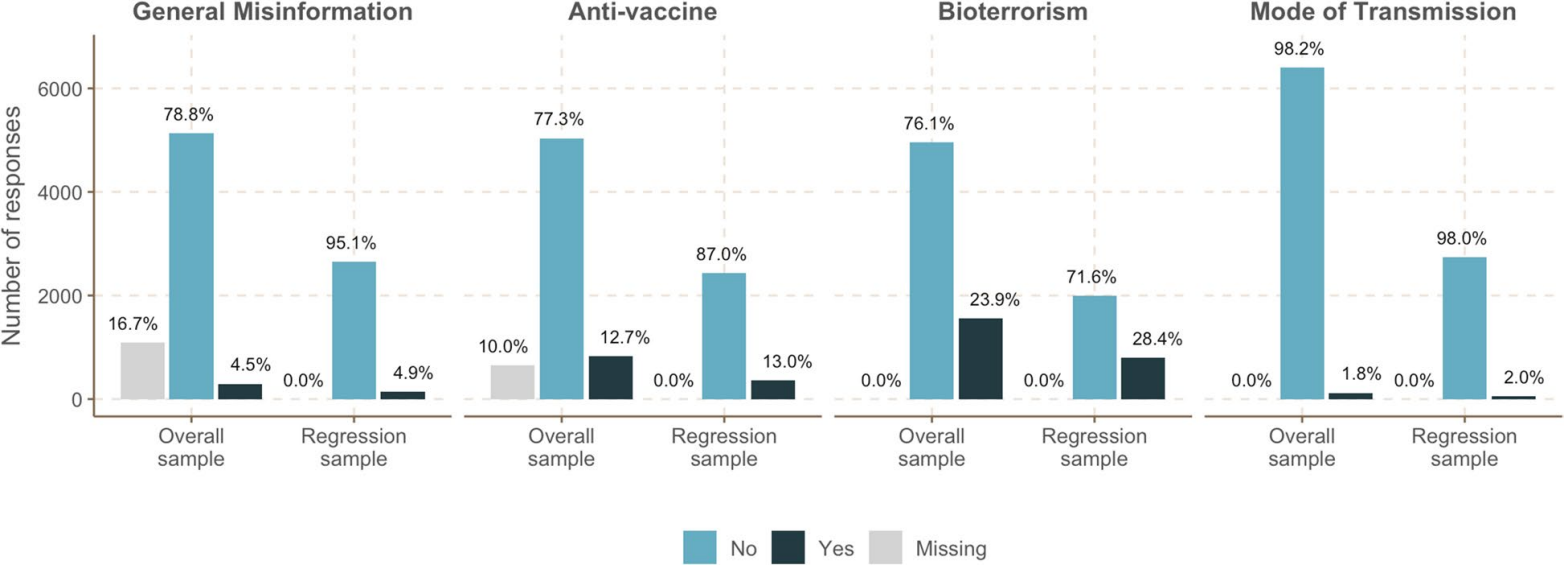
Distribution of survey participants believing in different types of misinformation in the total sample (*N* = 6518) and the regression sample (*N* = 2793)

Overall, 31.4% (*n* = 2048) of the total respondents and 35.2% (*n* = 982) of the respondents included in the regression sample believed in at least one type of misinformation. In the overall sample, 23.9% (*n* = 794) believed in bioterrorism misinformation, 12.7% (*n* = 826) believed in misinformation about a hypothetical COVID-19-vaccine, 4.5% (*n* = 294) of the respondents believed in general misinformation, and 1.8% (*n* = 120) believed misinformation about the mode of COVID-19 transmission. The proportion of people believing in misinformation was generally similar between the total and the regression sample, as shown in Fig. 1. Interestingly, belief in one type of misinformation was not strongly associated with belief in other types of misinformation. While the overall prevalence of belief in any type of misinformation was estimated to be 35.2% (*n* = 982) in the regression sample, only 8.8% (*n* = 246) of the participants believed in two types of misinformation, 2% (*n* = 55) believed in three types of misinformation, and 0.1% (*n* = 3) believed in all four types of misinformation (Additional file 3, Fig. S3–1). The strongest correlation (Pearson’s correlation coefficient = 0.32) was observed between the belief in misinformation related to the hypothetical COVID-19 vaccine and bioterrorism, followed by the relationship between belief in anti-vaccine misinformation and modes of transmission (coefficient = 0.25). Cross-tabulation of belief in different types of misinformation is provided in the supplementary material (Additional file 3, Table S3–1 ~ 7).

### LASSO logistic regressions

**Fig. 2:**
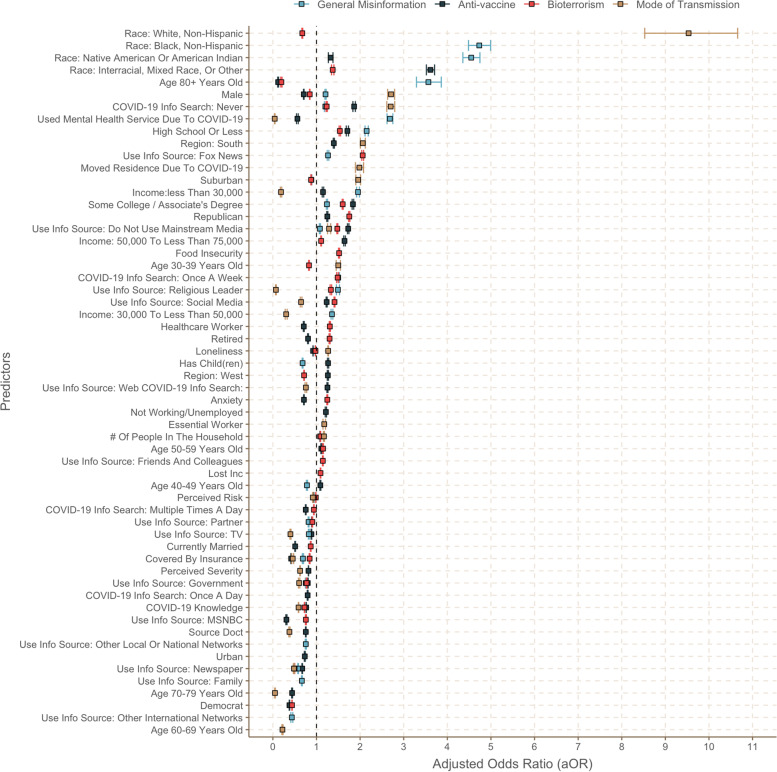
Factors associated with belief in four different types of COVID-19 misinformation (aggregated plot)

Figure 2 summarizes the results of the vector-bootstrapped LASSO logistic regression on the factors associated with belief in misinformation. A total of 58 factors were significantly associated with belief in at least one type of misinformation. Among them, 38 factors were positively associated and 38 were negatively associated with endorsement of at least one type of misinformation. Only two predictors, never searching COVID-19 information online and not using mainstream media as COVID-19 information source, were associated with significantly increased odds of believing in all four types of misinformation. Additionally, respondents’ highest educational attainment being high school or less or some college/associate’s degree predicted belief in three types of misinformation—general, anti-vaccine, and bioterrorism misinformation. Being Native American or American Indian, or of mixed race, male, Republican, a resident of the South or earning annual household income less than $30,000 was a common predictor for two different types of misinformation, as was using Fox News, a religious leader, or social media as a COVID-19 information source. Conversely, using a newspaper or the government’s official communication as a source of COVID-19-related information or having a health insurance coverage was associated with significantly lower odds of believing in all types of misinformation. Higher COVID-19-related knowledge or using TV as COVID-19 information source similarly predicted significantly lower odds of believing in misinformation related to the hypothetical COVID-19 vaccine, bioterrorism, and modes of transmission.

Interestingly, 18 predictors worked in opposite directions for different types of misinformation. For example, respondents’ age being 80 and above was a predictor for higher odds of believing in general misinformation, but was associated with lower odds of believing in bioterrorism or anti-vaccine misinformation. While those who used a mental health service due to COVID-19 reported higher odds of believing general misinformation, the use of a mental health service was associated with decreased odds of believing anti-vaccine or transmission mode misinformation. Similarly, people with high levels of anxiety, who are retired, or who reported to be a healthcare worker had higher odds of believing bioterrorism misinformation and decreased odds of believing in anti-vaccine misinformation.

**Fig. 3:**
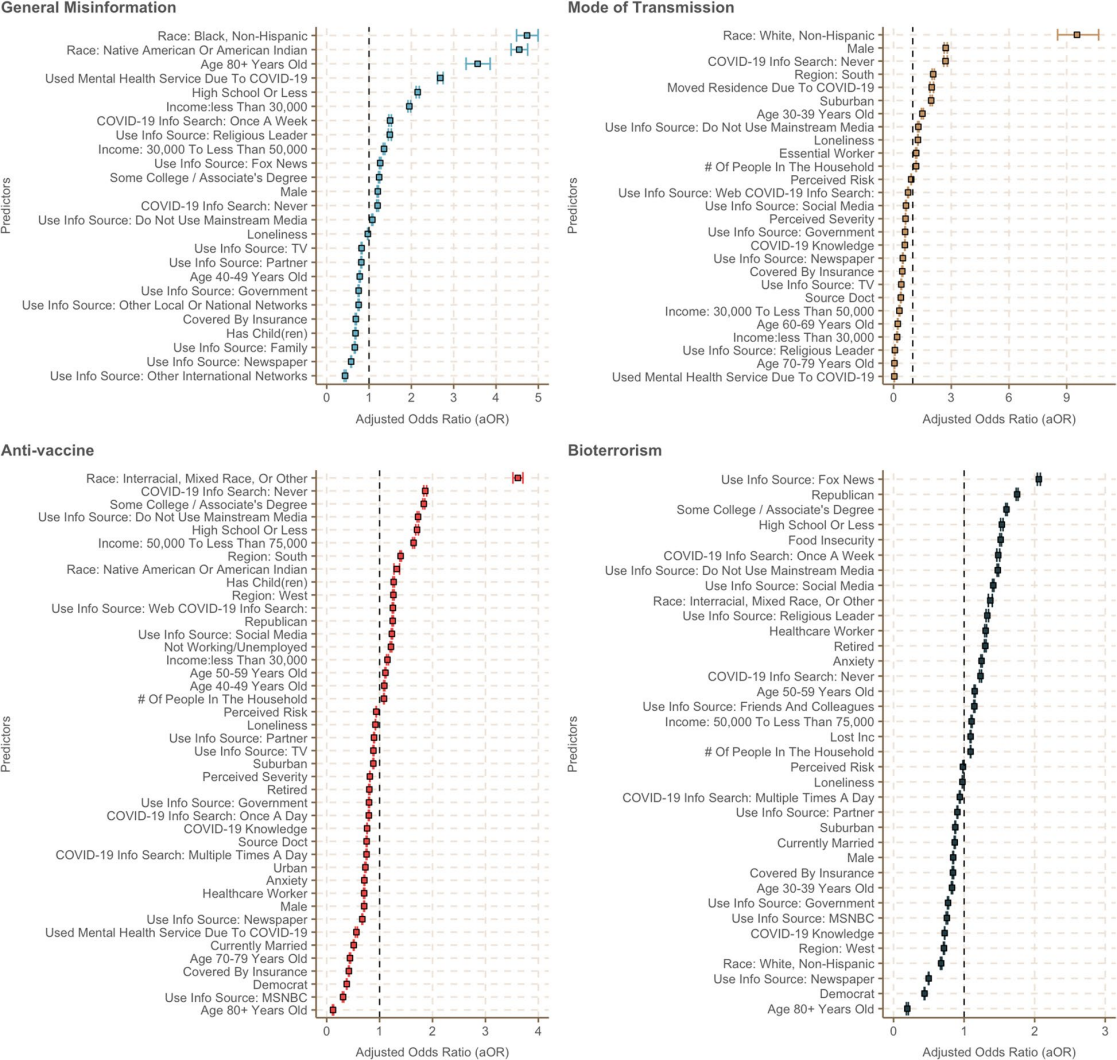
Factors associated with the belief in four different types of COVID-19 misinformation (expanded plot)

Figure 3 presents the predictors of each misinformation type in descending order by effect size. Respondents had higher odds of believing in general misinformation if they were Black, non-Hispanic or Native American/American Indian, aged 80 years and above, male, having highest educational attainment of high school degree or less, or earning less than $50,000 annual household income. Similarly, higher odds of believing in general misinformation was observed when they self-reported seeking mental health services for COVID-19, using Fox News as a COVID-19 information source, or never seeking COVID-19 information. Belief in anti-vaccine misinformation was most strongly associated with being mixed race or Native American/American Indian, never seeking COVID-19-related information, or lower educational attainment of some college or associate’s degree and below. Belief in bioterrorism misinformation was strongly and significantly associated with many predictors, including being politically affiliated with the Republican party, having low educational attainment of college or associate’s degree or less, being food insecure, and a user of Fox News, social media, or a religious leader as the primary COVID-19 information source. Finally, belief in misinformation on the mode of transmission was most strongly associated with never seeking COVID-19-related information, being male, having moved residence due to COVID-19, or reporting a higher level of loneliness. Summarized adjusted odds ratios and the 95% bootstrap CIs are available in the supplementary material (Additional file 4, Table S4–1 ~ 4).

## Discussion

Countering infodemics with targeted, factual information is crucial for ending the COVID-19 pandemic [1, 4]. Understanding what factors have played a role in people’s belief in COVID-19 misinformation is critical to enabling policymakers to craft strategic communications to manage the COVID-19 infodemic and may provide insights on how to tackle the future infodemics related to novel infectious disease threats. Our study attempted to identify the factors associated with belief in certain types of COVID-19 misinformation among US adults in April 2020 and showed that misinformation started affecting the general public from the early phase of the pandemic. We performed our analysis on four types of misinformation: general, anti-vaccine, bioterrorism, and transmission modes. Our use of LASSO regressions allowed us to identify and select significant predictors from a broad pool of potential factors while simultaneously reducing selection bias. This approach overcomes some of the limitations of the traditional approaches where a predetermined subset of predictors is used and constitutes a marked improvement by reducing the bias and improving the model’s robustness. Using bootstrapping, we further refined predictor selection and quantified our estimates’ standard error, which increased our confidence in our results.

First, we found that more than 30% of our sample of US adults on social media reported believing in at least one type of COVID-19-related misinformation in early 2020. This high prevalence highlights the importance of counter measures that can address the spread of misinformation to manage the infodemic. Second, we found that particular demographic and socioeconomic factors predicted respondents’ susceptibility to COVID-19 misinformation. Of the 66 variables included in our analysis, 58 were significantly associated with increased or decreased odds of believing in specific types of misinformation about COVID-19. Many of these variables were characteristics that are readily available and routinely collected as part of other national surveys, which suggests that policymakers could develop and leverage cost-effective predictive models using existing datasets to identify specific communities and individuals more likely to believe in misinformation.

Third, we found that different audiences were susceptible to different types of misinformation. The lack of strong correlation between beliefs in different types of misinformation is particularly interesting as it contradicts some prior literature that either showed strong positive associations between beliefs in mutually exclusive conspiracy theories [46] or described common psychological factors or mechanisms that promote people’s overall susceptibility to fake news in general [47, 48]. This observed difference may be explained by the existence of diverse psychological factors [48] that influence people’s tendency to fall for misinformation and their complex interactions with the environmental and sociodemographic factors, which calls for further research dedicated to this topic. Prior research on COVID-19 misinformation tended to aggregate all types of misinformation into a unified index despite the weak correlation between the types of misinformation [29]. This method overlooked key differences and made it difficult to identify differences between sociodemographic groups’ belief in misinformation, which in turn led to policymakers treating everyone who believes in any COVID-19 misinformation as one target group for interventions. As previously noted, anti-misinformation communication strategies need to be targeted to specific subgroups to be effective [14, 49]. Our findings confirmed that there are clear differences in subgroups’ belief in misinformation, and should be used to inform strategies that effectively engage those groups by understanding their existing beliefs and motivations, and that address the structural and economic factors that facilitate or promote belief in misinformation [14]. Furthermore, our study sets the stage for further research that investigates the association between the belief in specific type(s) of misinformation and various COVID-19-related health behaviors, which can inform effective intervention strategies against the transmission of disease.

Our study had several limitations. First, we used nonprobability convenience sampling via social media platforms affiliated with Facebook to collect our survey data. Because of this approach, our sample may not be representative of the US adult population, despite our sample’s large sample size (see Additional file 5, Table S5–1 for the detailed comparison) [32]. While our sample is balanced across geography, age groups, and other sociodemographic characteristics, we acknowledge the under-representation of certain subpopulations that might be particularly vulnerable to misinformation. For example, our choice of sampling platform systemically excluded people without access to the internet or social media platforms. Given time constraints and the impracticality of face-to-face recruitment due to the COVID-19 pandemic, we chose the social media platforms affiliated with Facebook as a recruitment and dissemination platform to maximize our reach to the general US population; 70% of the US population are estimated to have Facebook accounts, and among those with accounts, 75% use Facebook daily [50]. Foreign-born adults with limited English-speaking skills, comprising over 40 million adults [51], would additionally have been excluded from our sample. As a result, the participants in our study were overwhelmingly non-Latinx white despite our concerted effort to oversample potentially under-represented sociodemographic groups [32]. Thus, given these sampling limitations, the findings from our study cannot be generalized to the US population. In particular, those under-represented subpopulations may likely provide further insights into different subgroups who believe in misinformation. Several studies have highlighted immigrants’ elevated risk of exposure to misinformation and difficulty in accessing needed information and resources during the COVID-19 pandemic [52–54]. Further research focusing on this under-represented community is therefore warranted. On the other hand, convenience sampling through popular social media platforms might have resulted in recruiting a particular subpopulation that was more exposed to the COVID-19 infodemic, which mainly propagates through informal online sources [17], hence was more vulnerable to be misinformation. A recent study concluded that Facebook alone was accountable for over two thirds of the COVID-19 misinformation produced across all social media platforms during the first year of the pandemic [6]. In this regard, despite the non-representativeness of the sample, we believe that our study yielded meaningful insights based on the “information-rich” sample of this population with elevated exposure to misinformation.

Second, our regression analysis was conducted on a subset of the sample that only contains complete responses with no missing data. Despite that the completion time of the web-based survey was under 15 minutes and was deemed appropriate [55], the response rate was around 43%. While it is difficult to discern the reasons behind the missing responses as the patterns of missingness across survey questions is likely to depend on multiple factors [56, 57], the response rate to our survey is within the acceptable range compared to other web-based surveys for public health research [58–60]. Since most missing data were MCAR, we did not perform any imputations. Differential missingness of certain responses which were MAR and whose missingness is associated with the outcome variables [35, 61], however, might have caused some bias. Despite this limitation, we believe our findings still provide novel and significant insights on specific groups. Further, we believe that the benefits of our methodological approach—namely the LASSO regressions, which require complete data—outweigh the costs of subsetting our dataset.

Third, we note that our misinformation categories, which were formulated in the nascent stages of the pandemic, were not necessarily the categories of misinformation that ultimately played the most significant role in individuals’ belief in—or rejection of—public health guidelines. For instance, “bioterrorism” ultimately had less bearing on the public than other strains of misinformation, and anti-vaccine misinformation proliferated and became more nuanced after the first vaccines were released. The fact that 42 out of 58 demographic and socioeconomic factors were identified as important predictors of anti-vaccine misinformation may imply that this dependent variable consists of many sub-types of anti-vaccine misinformation that could be further broken down in granularity. Future research should seek to investigate specific strains of misinformation, such as “vaccine chip” misinformation versus “vaccine poison” misinformation.

Moreover, it is worth noting the current lack of validated and reliable approaches to define and measure the prevalence of COVID-19 misinformation. Due to this constraint, our study used a single survey item for each type of misinformation to define the four outcome variables, without means to test their validity and reliability. As a result, we cannot state with confidence that our chosen survey items are superior to other alternative approaches in defining these outcome variables. For example, our anti-vaccine variable was defined using the question “How likely would you be to get a Coronavirus vaccine if it was recommended by: doctor/medical provider?” while other studies used the question “Once a vaccine to prevent COVID-19 is available to you, would you get a vaccine?” [62] One may argue that both approaches may not be able to discern the true belief in misinformation arising from different underlying reasoning and understanding of the question. For example, people who suffer from needle phobia may answer “not likely” to both questions even when they do not believe in vaccine-related misinformation. However, with the current lack of a consensus and an agreed-upon approach, it is extremely challenging to argue which method would be most appropriate. Therefore, while our study method is consistent with the approaches taken by the existing studies exploring similar research questions [8, 27, 63], the aforementioned limitation strongly calls for the development of a set of instruments that can be used in COVID-19 misinformation research to improve the reliability and comparability of the findings. To be thorough, we repeated the analysis using alternative survey items to define six other outcome variables, five for anti-vaccine and one for transmission mode, and included the results in the supplementary material (Additional file 6).

Finally, we note that the number of respondents who believed in transmission mode misinformation was very small (*N* = 56), and that particularly in the US context, an underlying cultural lack of conformity with government mandates may have also contributed to individuals responding that they would not comply with government mandated social distancing. The transmission mode misinformation results should therefore be interpreted with some skepticism.

## Conclusions

The proliferation of user-generated content on social media has accelerated and perpetuated the spread of misinformation [1, 4]. Misinformation can play a significant role in misdirecting individuals’ decision making and belief in public health guidelines, which in turn hinders effective management and control of the COVID-19 pandemic. To effectively counter misinformation, communication strategies and messaging should be tailored to the targeted populations. Our findings provides policymakers with a more nuanced understanding of the different subgroups within the misinformed population. These subgroups must be targeted with different types of messages and strategies to improve the effectiveness of public health efforts to counter COVID-19 misinformation. For this, as a next step, policymakers may want to further categorize the identified predictors into several dimensions, using, for example, principal component analysis, in order to better understand their target audience and finetune their communication strategies to counter the infodemic. Improved specificity of interventions targeting at countering misinformation, when combined with other strategies that focus on reducing the generation of and the exposure of people to misinformation, could more effectively curb the infodemic. Our study further establishes research best practices in the early stages of an epidemic or pandemic, and demonstrates the use of a novel methodology to pinpoint belief in specific types of misinformation.

## Supplementary Information

**Additional file 1.** Complete questionnaire used in April 2020.

**Additional file 2.** Descriptive statistics.

**Additional file 3.** Cross-tabulation of belief in different types of misinformation.

**Additional file 4.** Summary of the predictors for COVID-19 misinformation, adjusted odds ratios, and their 95% bootstrap confidence interval.

**Additional file 5.**

**Additional file 6.**

## Data Availability

The datasets used and/or analysed during the current study are available from the corresponding author on reasonable request.
