# Supplementary material for: What predicts people’s belief in COVID-19 misinformation? A retrospective study using a nationwide online survey among adults residing in the United States
Source: BMC Public Health. 2022 Nov 18;22:2114. doi: 10.1186/s12889-022-14431-y (PMC9673212; doi:10.1186/s12889-022-14431-y)
Supplement: Supplementary file 1 — Additional file 1. Complete questionnaire used in April 2020. [file 12889_2022_14431_MOESM1_ESM.docx]

Supplementary Material 1

Table S1-1. Complete questionnaire used in the second wave of the survey (April 2020)

| **Code** | Description | Measurement |
| --- | --- | --- |
| **elig18** | Are you 18 years or older? | 0=No  1=Yes |
| **eligUS** | Do you currently reside in the US? | 0=No  1=Yes |
| **completed** | Dummy variable | 0=No (Did not complete among those who started the survey)  1=Completed 100% |
| **sex** | Sex | 0=Female  1=Male  2=Other  3=Prefer not to disclose |
| **age** | Age group | 0=18-29 years old  1= 30-39 years old  2= 40-49 years old  3=50-59 years old  4= 60-69 years old  5=70-79 years old  6=80+ years old |
| **covid_test** | Have you been tested for Coronavirus? | 0=No  1=Yes |
| **covid_try** | Have you tried to get tested for Coronavirus? | 0=No  1=Yes |
| **covid_able** | Do you think you would be able to get a test for Coronavirus if you thought you needed one? | 0=No  1=Yes |
| **covid_hosp** | Do you know anyone in your immediate social environment (including yourself) who has been hospitalized with Coronavirus? | 0=No  1=Yes |
| **insur** | Are you now covered by any form of health insurance or health plan? | 0=No  1=Yes |
| **insur_source** | Which of the following is your MAIN source of health insurance coverage? | 0=Plan through your employer  1=Plan through your spouse’s employer  2=Plan you purchased yourself  3=Medicare  4=Medicaid/State-specific Medicaid  5=Somewhere else  6=Paln through your parents/mother/father |
| **flu** | Since July 1st 2019, have you had a flu vaccination? | 0=No  1=Yes |
| **vacc_doct** | How likely would you be to get a Coronavirus vaccine if it was recommended by: doctor/medical provider | 0=Not likely  1=Somewhat likely  2=Very likely |
| **vacc_fre** | How likely would you be to get a Coronavirus vaccine if it was recommended by: friends | 0=Not likely  1=Somewhat likely  2=Very likely |
| **vacc_fam** | How likely would you be to get a Coronavirus vaccine if it was recommended by: family members | 0=Not likely  1=Somewhat likely  2=Very likely |
| **vacc_fed** | How likely would you be to get a Coronavirus vaccine if it was recommended by: federal government | 0=Not likely  1=Somewhat likely  2=Very likely |
| **vacc_relig** | How likely would you be to get a Coronavirus vaccine if it was recommended by: religious leaders | 0=Not likely  1=Somewhat likely  2=Very likely |
| **vacc_loc** | How likely would you be to get a Coronavirus vaccine if it was recommended by: local health department | 0=Not likely  1=Somewhat likely  2=Very likely |
| **avail_imm** | If a Coronavirus vaccine became AVAILABLE, how confident are you that you would: Try to get the Coronavirus vaccine immediately | 0=Not likely  1=Somewhat likely  2=Very likely |
| **avail_pay** | If a Coronavirus vaccine became AVAILABLE, how confident are you that you would: Pay for the vaccine out of pocket | 0=Not likely  1=Somewhat likely  2=Very likely |
| **getvacc_knew** | How likely would you be to get a Coronavirus vaccine if: Someone you knew became sick or died from Coronavirus. | 0=Not likely  1=Somewhat likely  2=Very likely |
| **getvacc_neigh** | How likely would you be to get a Coronavirus vaccine if: Many in your neighborhood became sick or died from Coronavirus. | 0=Not likely  1=Somewhat likely  2=Very likely |
| **getvacc_symp** | How likely would you be to get a Coronavirus vaccine if: There’s a chance you will develop mild side effects (like a cold) from the vaccine. | 0=Not likely  1=Somewhat likely  2=Very likely |
| **getvacc_70** | How likely would you be to get a Coronavirus vaccine if: The vaccine was 70% effective in preventing infection. | 0=Not likely  1=Somewhat likely  2=Very likely |
| **getvacc_90** | How likely would you be to get a Coronavirus vaccine if: The vaccine was 90% effective in preventing infection. | 0=Not likely  1=Somewhat likely  2=Very likely |
| **opinvacc_right** | Vaccinations are a right that we should all have access to. | 0=FALSE  1=TRUE |
| **opinvacc_free** | Vaccinations can help you be free to live a happy and healthy life. | 0=Never  1=Often  2=Rarely  3=Sometimes |
| **lon_1** | In the last 3 months, how often have you felt: Left out | 0=Never  1=Often  2=Rarely  3=Sometimes |
| **lon_2** | In the last 3 months, how often have you felt: Isolated from others | 0=Never  1=Often  2=Rarely  3=Sometimes |
| **lon_3** | In the last 3 months, how often have you felt: That you lack companionship | 0=Never  1=Often  2=Rarely  3=Sometimes |
| **lost_inc** | Have you lost income from a job or business because of the coronavirus? | 0=No  1=Yes  2=Not applicable |
| **workworry_inc** | How worried, if at all, are you that: You will lose income due to a workplace closure or reduced hours because of Coronavirus? | 0=Not at all worried  1=Not too worried  2=Somewhat worried  3=Very worried  4=Not applicable |
| **workworry_inv** | How worried, if at all, are you that: Your investments such as retirement or college savings will be negatively impacted by Coronavirus? | 0=Not at all worried  1=Not too worried  2=Somewhat worried  3=Very worried  4=Not applicable |
| **workworry_risk** | How worried, if at all, are you that: You will put yourself at risk of exposure to Coronavirus because you can’t afford to stay home and miss work? | 0=Not at all worried  1=Not too worried  2=Somewhat worried  3=Very worried  4=Not applicable |
| **workworry_tre** | How worried, if at all, are you that: You will not be able to afford treatment for Coronavirus if you need it? | 0=Not at all worried  1=Not too worried  2=Somewhat worried  3=Very worried  4=Not applicable |
| **food_1** | In the last 3 months: The food that (I/we) bought just didn’t last, and (I/we) didn’t have money to get more. | 0=Never True  1=Sometimes True  2=Often True  3=Don’t know/Prefer not to say |
| **food_2** | In the last 3 months: (I/we) couldn’t afford to eat balanced meals. | 0=Never True  1=Sometimes True  2=Often True  3=Don’t know/Prefer not to say |
| **food_3** | In the last 3 months: Because I need food to eat, I have eaten packaged foods that have expired dates or that are passed the "best by" dates. | 0=Never True  1=Sometimes True  2=Often True  3=Don’t know/Prefer not to say |
| **food_4** | In the last 3 months: Because I need food to eat, I have eaten perishable food such as fruits and vegetables that do not appear to be fresh. | 0=Never True  1=Sometimes True  2=Often True  3=Don’t know/Prefer not to say |
| **food_5** | In the last 3 months, did you or other adults in your household ever cut the size of your meals or skip meals because there wasn't enough money for food? | 0=No  1=Yes |
| **food_6** | How often did this happen? [linked with food_5] | 0=Only 1 or 2 weeks  1=Some weeks but not every week  2=Almost every week  3=Don’t know |
| **food_7** | In the last 3 months: Did you ever eat less than you felt you should because there wasn't enough money for food? | 0=No  1=Yes  2=Don’t know |
| **food_8** | In the last 3 months: Were you ever hungry but didn't eat because there wasn't enough money for food? | 0=No  1=Yes  2=Don’t know |
| **know_1** | Coronavirus is a contagious disease. | 0=FALSE  1=TRUE |
| **know_2** | A person infected with Coronavirus is not contagious until after symptoms appear. | 0=FALSE  1=TRUE |
| **know_3** | Coronavirus cannot be spread through sneezing and coughing. | 0=FALSE  1=TRUE |
| **know_4** | Currently, there is an FDA approved drug for treating individuals with Coronavirus. | 0=FALSE  1=TRUE |
| **know_5** | Coronavirus can live on surfaces outside of the body for a few hours or several days. | 0=FALSE  1=TRUE |
| **know_6** | There is no vaccine currently available to prevent infection with Coronavirus. | 0=FALSE  1=TRUE |
| **know_7** | Children are at high risk for complications from Coronavirus. | 0=FALSE  1=TRUE |
| **know_8** | Older people with other health conditions are more likely to die from Coronavirus. | 0=FALSE  1=TRUE |
| **know_9** | People with Coronavirus can have no symptoms at all. | 0=FALSE  1=TRUE |
| **know_10** | Most people with Coronavirus will have severe or critical symptoms. | 0=FALSE  1=TRUE |
| **know_11** | Alcohol-based hand sanitizers cannot protect you from Coronavirus. | 0=FALSE  1=TRUE |
| **know_12** | Coronavirus may be transmitted by mosquito bites. | 0=FALSE  1=TRUE |
| **know_13** | Coronavirus originated from animals | 0=FALSE  1=TRUE |
| **know_14** | Animals can contract Coronavirus | 0=FALSE  1=TRUE |
| **knowinf_1** | Washing your hands frequently with soap and water. | 0=FALSE  1=TRUE |
| **knowinf_2** | Getting a flu shot. | 0=FALSE  1=TRUE |
| **knowinf_3** | Wearing a face mask. | 0=FALSE  1=TRUE |
| **knowinf_4** | Stop going to school/work. | 0=FALSE  1=TRUE |
| **knowinf_5** | Wiping potentially contaminated surfaces with a disinfectant. | 0=FALSE  1=TRUE |
| **knowinf_6** | Staying away from Asian people. | 0=FALSE  1=TRUE |
| **knowinf_7** | Staying away from people who sneeze and cough. | 0=FALSE  1=TRUE |
| **knowinf_8** | Avoiding touching your eyes, nose and mouth. | 0=FALSE  1=TRUE |
| **knowinf_9** | Taking antibiotics. | 0=FALSE  1=TRUE |
| **knowinf_10** | Stop eating Chinese food. | 0=FALSE  1=TRUE |
| **house** | How many people currently live in your household including yourself? | 1=1  2=2  3=3  4=4  5=5  6=6 or more |
| **resid_covid** | Have you been staying at your primary residence since the Coronavirus outbreak began? | 0=Yes  1=No, I traveled to stay with family or friends to quarantine together  2=No, I traveled to a second residence/vacation home to quarantine  3=No, I am staying at a secondary location to avoid exposing my family or roommates  4=Other |
| **search** | In the last week, how often have you searched for information about Coronavirus? | 0=Never  1=Once a week  2=A couple of times a week  3=Once a day  4=Multiple times a day |
| **source_part** | Do you get Coronavirus information from:  Spouse/Partner | 0=No  1=Yes  2=Not applicable |
| **source_fam** | Do you get Coronavirus information from:  Other family members | 0=No  1=Yes  2=Not applicable |
| **source_fre** | Do you get Coronavirus information from:  Friends or Coworkers | 0=No  1=Yes  2=Not applicable |
| **source_reli** | Do you get Coronavirus information from:  Religious leader (e.g., minister) | 0=No  1=Yes  2=Not applicable |
| **source_doct** | Do you get Coronavirus information from:  Doctor/medical provider | 0=No  1=Yes  2=Not applicable |
| **source_tv** | Do you get Coronavirus information from:  TV | 0=No  1=Yes  2=Not applicable |
| **source_radi** | Do you get Coronavirus information from:  Radio or Podcasts | 0=No  1=Yes  2=Not applicable |
| **source_news** | Do you get Coronavirus information from:  Newspaper (printed or internet, e.g., the New York Times) | 0=No  1=Yes  2=Not applicable |
| **source_gov** | Do you get Coronavirus information from:  Government or other official websites (e.g., the CDC or WHO) | 0=No  1=Yes  2=Not applicable |
| **source_soc** | Do you get Coronavirus information from:  Social media (e.g., Facebook or Twitter) | 0=No  1=Yes  2=Not applicable |
| **source_web** | Do you get Coronavirus information from:  Google search, Wikipedia or other non-government websites | 0=No  1=Yes  2=Not applicable |
| **mainst_** | Do you seek information on Coronavirus from mainstream media sources (e.g., CNN, Fox News, MSNBC, Local/national networks, International networks)? | 0=No  1=Yes |
| **mainst_most** | Which of the below mainstream media sources do you get the most information from? | 0=CNN  1=Fox news  2=MSNBC  3=Other local or national networks  4=Other International networks (e.g., BBC, Al Jazeera, Sky News) |
| **source_most** | Which information source below do you trust the most for Coronavirus (select one)? | 0=Spouse/Partner  1=Other family members  2=Friends or Coworkers  3=Religious leader (e.g., minister)  4=Doctor/medical provider  5=TV  6=Radio or Podcasts  7=Newspaper (printed or internet, e.g., the New York Times)  8= Government or other official websites (e.g., the CDC or WHO)  9=Social media (e.g., Facebook or Twitter)  10=Google search; Wikipedia or other non-government websites |
| **info_accu** | I believe the information I get about Coronavirus is accurate. | 0=No  1=Yes |
| **info_upto** | I believe the information I get about Coronavirus is up-to-date. | 0=No  1=Yes |
| **behav_1** | Got a flu shot (or had my children get a flu shot) after hearing about Coronavirus. | 0=FALSE  1=TRUE |
| **behav_2** | Purchased a face mask. | 0=FALSE  1=TRUE |
| **behav_3** | Started working from home. | 0=FALSE  1=TRUE |
| **behav_4** | Started using hand-sanitizer and/or washing my hands more often. | 0=FALSE  1=TRUE |
| **behav_5** | Started drinking more fluids and/or getting more rest. | 0=FALSE  1=TRUE |
| **behav_6** | Started taking antiviral and/or antibiotics. | 0=FALSE  1=TRUE |
| **behav_7** | Started taking dietary supplements (e.g., vitamins, probiotics). | 0=FALSE  1=TRUE |
| **behav_8** | Avoided using public transportation. | 0=FALSE  1=TRUE |
| **behav_9** | Kept away from crowded places. | 0=FALSE  1=TRUE |
| **behav_10** | Started cleaning and/or disinfecting things that I might touch (e.g., doorknobs, phone). | 0=FALSE  1=TRUE |
| **behav_11** | Started wearing rubber gloves in public. | 0=FALSE  1=TRUE |
| **behav_12** | Started taking more hot baths. | 0=FALSE  1=TRUE |
| **behav_13** | Started spending more time at home | 0=FALSE  1=TRUE |
| **behav_14** | Started wearing a face mask or cloth face covering when I leave home | 0=FALSE  1=TRUE |
| **behav_15** | Started practicing social distancing | 0=FALSE  1=TRUE |
| **behav_16** | Avoided seeking medical or dental care for other health concerns | 0=FALSE  1=TRUE |
| **behav_17** | Avoided leaving home except for food or medical supplies | 0=FALSE  1=TRUE |
| **behav7_1** | Past 7 days because of Coronavirus I have: Maintained a stockpile of food and/or water. | 0=No  1=Yes |
| **behav7_2** | Past 7 days because of Coronavirus I have: Cancelled/postponed a social event (e.g., meeting friends, eating out, going to sports events). | 0=No  1=Yes |
| **behav7_3** | Past 7 days because of Coronavirus I have: Discussed with a friend or family member what we would do if one of us catches coronavirus. | 0=No  1=Yes |
| **behav7_4** | Past 7 days because Coronavirus I have: Stayed away from individuals at risk for severe Coronavirus (e.g., the elderly). | 0=No  1=Yes |
| **smoke** | Since Coronavirus, has behavior changed:  Smoking (tobacco products) | 0=Much less  1=Little less  2=Not changed  3=Little more  4=Much more  5=Not applicable |
| **vape** | Since Coronavirus, has behavior changed:  Vaping or e-cigarettes | 0=Much less  1=Little less  2=Not changed  3=Little more  4=Much more  5=Not applicable |
| **drink** | Since Coronavirus, has behavior changed:  Drinking (alcohol) | 0=Much less  1=Little less  2=Not changed  3=Little more  4=Much more  5=Not applicable |
| **sleep** | Since Coronavirus, has behavior changed:  Sleeping | 0=Much less  1=Little less  2=Not changed  3=Little more  4=Much more  5=Not applicable |
| **exerc** | Since Coronavirus, has behavior changed:  Exercising | 0=Much less  1=Little less  2=Not changed  3=Little more  4=Much more  5=Not applicable |
| **eat** | Since Coronavirus, has behavior changed:  Eating | 0=Much less  1=Little less  2=Not changed  3=Little more  4=Much more  5=Not applicable |
| **trav_sev** | Until Coronavirus is controlled, I would not:  Travel to any of the countries severely affected by Coronavirus. | 0=Strongly Disagree  1=Disagree  2=Agree  3=Strongly Agree |
| **trav_cruise** | Until Coronavirus is controlled, I would not:  Take a cruise | 0=Strongly Disagree  1=Disagree  2=Agree  3=Strongly Agree |
| **trav_plan_int** | Until Coronavirus is controlled, I would not:  Travel by plane internationally | 0=Strongly Disagree  1=Disagree  2=Agree  3=Strongly Agree |
| **trav_plan_dom** | Until Coronavirus is controlled, I would not:  Travel by plane within the United States | 0=Strongly Disagree  1=Disagree  2=Agree  3=Strongly Agree |
| **percv_risk** | On a scale from 0-10, what do you think is your risk of getting infected with Coronavirus? | Scale: 0 (Not at all likely) to 10 (Extremely likely) |
| **mon_cases** | In the NEXT 3 MONTHS, how many people in the United States do you think will contract the Coronavirus? | 0=Up to 100  1=Up to 1,000  2=Up to 10,000  3=Up to 100,000  4=Up to 1,000,000  5=Up to 10,000,000  6=More than 10,000,000 |
| **prop_cases** | By August 2020, what proportion of the population of the United States do you think will have been infected by Coronavirus? | 0=Fewer than 5%  1=Between 6% and 19%  2=Between 20% and 39%  3=Between 40% and 59%  4=Between 60% and 79%  5=Between 80% and 100% |
| **percv_sev** | On a scale from 0-10, If you were infected with Coronavirus, how severe do you think it would be? | Scale: 0 (Not Severe) to 10 (Very Severe) |
| **belief_1** | If I were ORDERED to quarantine myself due to Coronavirus, I would do so. | 0=Strongly Disagree  1=Disagree  2=Agree  3=Strongly Agree |
| **belief_2** | If I were ASKED to self-quarantine due to Coronavirus, I would do so. | 0=Strongly Disagree  1=Disagree  2=Agree  3=Strongly Agree |
| **belief_3** | I can financially afford to self-quarantine. | 0=Strongly Disagree  1=Disagree  2=Agree  3=Strongly Agree |
| **belief_4** | Since the Coronavirus outbreak, I feel discriminated against. | 0=Strongly Disagree  1=Disagree  2=Agree  3=Strongly Agree |
| **belief_5** | Since the Coronavirus outbreak, others are staying away from me. | 0=Strongly Disagree  1=Disagree  2=Agree  3=Strongly Agree |
| **belief_6 (1)** | I think that Coronavirus was released as an act of bioterrorism. | 0=Strongly Disagree  1=Disagree  2=Agree  3=Strongly Agree |
| **belief_7 (2)** | Coronavirus is more deadly than the seasonal flu. | 0=Strongly Disagree  1=Disagree  2=Agree  3=Strongly Agree |
| **belief_8 (4)** | The amount of media attention devoted to Coronavirus has been adequate. | 0=Strongly Disagree  1=Disagree  2=Agree  3=Strongly Agree |
| **belief_9 (5)** | Coronavirus is not as big of a problem as the media suggests. | 0=Strongly Disagree  1=Disagree  2=Agree  3=Strongly Agree |
| **belief_10 (6)** | Coronavirus is a bigger problem than the government suggests. | 0=Strongly Disagree  1=Disagree  2=Agree  3=Strongly Agree |
| **belief_11 (3)** | I think warmer weather will reduce the spread of Coronavirus. | 0=Strongly Disagree  1=Disagree  2=Agree  3=Strongly Agree |
| **belief_12** | I'm more aware of my race/ethnicity when I'm in public due to the Coronavirus outbreak. | 0=Strongly Disagree  1=Disagree  2=Agree  3=Strongly Agree |
| **fedgov_resp** | On a scale from 0-10, How prepared is the U.S. federal government to handle the Coronavirus outbreak? | Scale: 0 (Totally unprepared) to 10 (Extremely prepared) |
| **statgov_resp** | On a scale from 0-10, How prepared is my state government to handle the Coronavirus outbreak? | Scale: 0 (Totally unprepared) to 10 (Extremely prepared) |
| **threat_secur** | The Coronavirus outbreak is a threat to our national security. | 0=Strongly Disagree  1=Disagree  2=Agree  3=Strongly Agree |
| **threat_econ** | The Coronavirus outbreak is a threat to our national economy. | 0=Strongly Disagree  1=Disagree  2=Agree  3=Strongly Agree |
| **threat_pers** | The Coronavirus outbreak is a threat to my personal finances. | 0=Strongly Disagree  1=Disagree  2=Agree  3=Strongly Agree |
| **attid_pol_1** | The United States should assume worldwide leadership in confronting the Coronavirus outbreak. | 0=Strongly Disagree  1=Disagree  2=Agree  3=Strongly Agree |
| **attid_pol_2** | The United States should assist other countries in confronting the Coronavirus outbreak by providing financial/medical resources. | 0=Strongly Disagree  1=Disagree  2=Agree  3=Strongly Agree |
| **attid_pol_3** | The United States should increase domestic funding for Coronavirus related medical resources and research. | 0=Strongly Disagree  1=Disagree  2=Agree  3=Strongly Agree |
| **attid_pol_4** | People who travel to countries where Coronavirus has been detected should be tested for Coronavirus before being allowed to return to the United States. | 0=Strongly Disagree  1=Disagree  2=Agree  3=Strongly Agree |
| **attid_pol_5** | People who travel to countries where the Coronavirus has been detected should be asked to quarantine for 14 days in the United States, even if they test negative for Coronavirus. | 0=Strongly Disagree  1=Disagree  2=Agree  3=Strongly Agree |
| **attid_pol_6** | The United States should ban the entry of individuals arriving from countries highly affected by the Coronavirus. | 0=Strongly Disagree  1=Disagree  2=Agree  3=Strongly Agree |
| **attid_pol_7** | The United States should ban the entry of any individual arriving from a foreign country until the Coronavirus outbreak is controlled. | 0=Strongly Disagree  1=Disagree  2=Agree  3=Strongly Agree |
| **optimism** | I am optimistic that the Coronavirus outbreak will be controlled in the next 3 months. | 0=Strongly Disagree  1=Disagree  2=Agree  3=Strongly Agree |
| **vote_?** | Are you eligible to vote in local, state, or federal elections in the United States? | 0=No  1=Yes |
| **vote_1** | Will you consider candidates’ policies on the Coronavirus outbreak when voting in the 2020 elections? | 0=No  1=Yes |
| **vote_2** | Do you think the Coronavirus outbreak will influence who gets elected in the 2020 elections? | 0=No  1=Yes |
| **vote_3** | Will you skip in-person voting if Coronavirus is spreading in your community? | 0=No  1=Yes |
| **vote_4** | Do you think that all people should be allowed to vote by mail (i.e., not in-person voting) if Coronavirus is spreading in the United States? | 0=No  1=Yes |
| **anx_1** | Feeling nervous, anxious, or on edge? | 0=Not at all  1=Several days  2=More than half the days  3=Nearly every day |
| **anx_2** | Not being able to stop or control worrying? | 0=Not at all  1=Several days  2=More than half the days  3=Nearly every day |
| **anx_3** | Feeling down, depressed, or hopeless? | 0=Not at all  1=Several days  2=More than half the days  3=Nearly every day |
| **anx_4** | Little interest or pleasure in doing things (that I used to enjoy)? | 0=Not at all  1=Several days  2=More than half the days  3=Nearly every day |
| **prob_time** | During the past 4 weeks, have you had any of the following problems with your work or other regular daily activities as a result of any emotional problems (such as feeling depressed or anxious)?: Cut down the amount of time you spent on work or other activities. | 0=No  1=Yes |
| **prob_acco** | During the past 4 weeks, have you had any of the following problems with your work or other regular daily activities as a result of any emotional problems (such as feeling depressed or anxious)?: Accomplished less than you would like. | 0=No  1=Yes |
| **prob_care** | During the past 4 weeks, have you had any of the following problems with your work or other regular daily activities as a result of any emotional problems (such as feeling depressed or anxious)?: Didn't do work or other activities as carefully as usual. | 0=No  1=Yes |
| **impact_1** | I thought about Coronavirus when I didn’t mean to. | 0=Not at all  1=Several days  2=More than half the days  3=Nearly every day |
| **impact_2** | I felt watchful or on-guard. | 0=Not at all  1=Several days  2=More than half the days  3=Nearly every day |
| **impact_3** | Other things kept making me think about Coronavirus. | 0=Not at all  1=Several days  2=More than half the days  3=Nearly every day |
| **impact_4** | I was aware that I still had a lot of feelings about Coronavirus, but I didn’t deal with them. | 0=Not at all  1=Several days  2=More than half the days  3=Nearly every day |
| **impact_5** | I tried not to think about Coronavirus. | 0=Not at all  1=Several days  2=More than half the days  3=Nearly every day |
| **impact_6** | I had trouble concentrating. | 0=Not at all  1=Several days  2=More than half the days  3=Nearly every day |
| **impactnew_1** | I thought about Coronavirus when I didn’t mean to. | 0=Not at all  1=A little bit  2=Moderately  3=Quite a bit  4=Extremely |
| **impactnew_2** | I felt watchful or on-guard. | 0=Not at all  1=A little bit  2=Moderately  3=Quite a bit  4=Extremely |
| **impactnew_3** | Other things kept making me think about Coronavirus. | 0=Not at all  1=A little bit  2=Moderately  3=Quite a bit  4=Extremely |
| **impactnew_4** | I was aware that I still had a lot of feelings about Coronavirus, but I didn’t deal with them. | 0=Not at all  1=A little bit  2=Moderately  3=Quite a bit  4=Extremely |
| **impactnew_5** | I tried not to think about Coronavirus. | 0=Not at all  1=A little bit  2=Moderately  3=Quite a bit  4=Extremely |
| **impactnew_6** | I had trouble concentrating. | 0=Not at all  1=A little bit  2=Moderately  3=Quite a bit  4=Extremely |
| **mental_serv** | Because of the Coronavirus outbreak, I have sought mental health services. | 0=No  1=Yes |
| **race** | What’s your race/ethnicity? | 0=White, Non-Hispanic  1=Black, Non-Hispanic  2=Hispanic/Latinx  3=Asian/Pacific Islander  4=Native American or American Indian  5=Interracial, Mixed race, or Other |
| **marit** | What is your marital or cohabitation status? | 0=Married/cohabiting  1=Single  2=Widowed  3=Divorced/separated |
| **state** | In which state do you currently reside? | 0=Alabama  1=Alaska  2=Arizona  3=Arkansas  4=California  5=Colorado  6=Connecticut  7=Delaware  8=Florida  9=Georgia  10=Hawaii  11=Idaho  12=Illinois  13=Indiana  14=Iowa  15=Kansas  16=Kentucky  17=Louisiana  18=Maine  19=Maryland  20=Massachusetts  21=Michigan  22=Minnesota  23=Mississippi  24=Missouri  25=Montana  26=Nebraska  27=Nevada  28=New Hampshire  29=New Jersey  30=New Mexico  31=New York  32=North Carolina  33=North Dakota  34=Ohio  35=Oklahoma  36=Oregon  37=Pennsylvania  38=Rhode Island  39=South Carolina  40=South Dakota  41=Tennessee  42=Texas  43=Utah  44=Vermont  45=Virginia  46=Washington  47=West Virginia  48=Wisconsin  49=Wyoming  50=Puerto Rico |
| **urban** | How would you describe where you live? | 0=Suburban  1=Urban  2=Rural |
| **work** | What is your current working status? | 0=Employed  1=Military personnel  2=Out of work and looking for work  3=Out of work but not currently looking for work  4=Retired  5=Self-employed  6=Student  7=Unable to work  8=Unpaid work (e.g., homemaker, eldercare, childcare) |
| **worknew** | What is your current working status? | 0=Employed, full time  1=Employed, part time  2=Military personnel  3=Out of work and looking for work  4=Out of work but not currently looking for work  5=Retired  6=Self-employed  7=Student  8=Unable to work  9=Unpaid work (e.g., homemaker, eldercare, childcare) |
| **work_wfh** | If you were required to remain at home because of a quarantine or work closure, would you be able to do at least part of your job from home? | 0=No  1=Yes  2=Not applicable |
| **work_sick** | Does your employer offer you paid time off if you are sick or ill? | 0=No  1=Yes  2=Not applicable |
| **work_sickfam** | Does your employer offer you paid time off to care for a family member who is sick or ill? | 0=No  1=Yes  2=Not applicable |
| **work_paytype** | Which of the following best describes how you get paid at work--you are paid an hourly rate, you are paid a salary, or you are paid by the job? | 0=Hourly rate  1=Salary  2=By the job  3=Other |
| **work_essent** | Are you considered an essential worker (ie. do you have to go in to work when others in your community have been asked to stay at home)? | 0=No  1=Yes |
| **healthcare** | Do you work in an environment where you come into contact with sick people in a healthcare or clinical setting (e.g., doctor, nurse, hospital staff, paramedic, etc.) | 0=No  1=Yes |
| **child** | Do children under 18 year old live in your household? | 0=No  1=Yes |
| **educ** | What is your highest level of education? | 0=Less than a Highschool diploma  1= High School diploma or GED  2= Some college  3=Bachelor’s degree  4= Masters/Professional degree or above |
| **educnew** | What is your highest level of education? | 0=Less than a Highschool diploma  1= High School diploma or GED  2= Some college, no degree  3=Associate's degree  4=Bachelor’s degree  5= Masters/Professional degree or above |
| **polit** | What is your political affiliation? | 0=Democrat  1=Republican  2=Other  3=Prefer not to say |
| **income** | Last year – that is, in 2019 – what was your total household income from all sources, before taxes? | 0=Less than $20,000  1=$20,000 to less than $30,000  2=$30,000 to less than $40,000  3=$40,000 to less than $50,000  4=$50,000 to less than $75,000  5=$75,000 to less than $90,000  6=$90,000 to less than $100,000  7=$100,000 or more  8=Don’t know  9=Prefer not to say |

GREEN – Items removed from the second wave (April 2020)

BLUE – Newly introduced in the second wave (April 2020)
