# Supplementary material for: What predicts people’s belief in COVID-19 misinformation? A retrospective study using a nationwide online survey among adults residing in the United States
Source: BMC Public Health. 2022 Nov 18;22:2114. doi: 10.1186/s12889-022-14431-y (PMC9673212; doi:10.1186/s12889-022-14431-y)
Supplement: Supplementary file 2 — Additional file 2. Descriptive statistics. [file 12889_2022_14431_MOESM2_ESM.docx]

Supplementary Material 2: Descriptive statistics

Table S2. Complete list of variables used in the analysis with their definition and the summary of participants’ response

|  | **Total sample (N=6,518)** | **Regression Sample (N=2,793)** |
| --- | --- | --- |
| **Sex** |  |  |
| Female | 3717 (57.0%) | 1610 (57.6%) |
| Male | 2738 (42.0%) | 1183 (42.4%) |
| Missing | 63 (1.0%) |  |
| **Age group** |  |  |
| 18-29 years old | 343 (5.3%) | 120 (4.3%) |
| 30-39 years old | 735 (11.3%) | 372 (13.3%) |
| 40-49 years old | 997 (15.3%) | 495 (17.7%) |
| 50-59 years old | 1814 (27.8%) | 863 (30.9%) |
| 60-69 years old | 1967 (30.2%) | 755 (27.0%) |
| 70-79 years old | 605 (9.3%) | 179 (6.4%) |
| 80+ years old | 57 (0.9%) | 9 (0.3%) |
| **Race** |  |  |
| White, Non-Hispanic | 6012 (92.2%) | 2634 (94.3%) |
| Hispanic/Latinx | 169 (2.6%) | 52 (1.9%) |
| Interracial, Mixed race, or Other | 190 (2.9%) | 63 (2.3%) |
| Asian/Pacific Islander | 50 (0.8%) | 15 (0.5%) |
| Black, Non-Hispanic | 53(0.8%) | 12 (0.4%) |
| Native American or American Indian | 44 (0.7%) | 17 (0.6%) |
| **Currently married** |  |  |
| No | 1475 (22.6%) | 492 (17.6%) |
| Yes | 3585 (55.0%) | 2301 (82.4%) |
| Missing | 1458 (22.4%) |  |
| **Children under 18 in the household** |  |  |
| No | 4253 (65.3%) | 1893 (67.8%) |
| Yes | 1477 (22.7%) | 900 (32.2%) |
| Missing | 788 (12.1%) |  |
| **Number of people in the household** |  |  |
| Mean (SD) | 3.16 (1.70) | 2.84 (1.26) |
| **Employment status** |  |  |
| Employed | 2845 (43.6%) | 1832 (65.6%) |
| Student/Unpaid work | 280 (4.3%) | 140 (5.0%) |
| Not working/Unemployed | 635 (9.7%) | 325 (11.6%) |
| Retired | 1300 (19.9%) | 496 (17.8%) |
| Missing | 1458 (22.4%) |  |
| **Highest educational attainment** |  |  |
| High school or less | 516 (7.9%) | 264 (9.5%) |
| Some college / Associate's degree | 1720 (26.4%) | 944 (33.8%) |
| Bachelor's degree or higher | 2792 (42.8%) | 1585 (56.7%) |
| Missing | 1490 (22.9%) |  |
| **Annual household income** |  |  |
| Less than $30,000 | 580 (8.9%) | 233 (8.3%) |
| $30,000 to less than $50,000 | 671 (10.3%) | 378 (13.5%) |
| $50,000 to less than $75,000 | 767 (11.8%) | 477 (17.1%) |
| $75,000 to less than $100,000 | 900 (13.8%) | 614 (22.0%) |
| $100,000 or more | 1419 (21.8%) | 1091 (39.1%) |
| Missing | 2181 (33.5%) |  |
| **Democrat (political affiliation)** |  |  |
| No | 3103 (47.6%) | 1716 (61.4%) |
| Yes | 1925 (29.5%) | 1077 (38.6%) |
| Missing | 1490 (22.9%) |  |
| **Republican (political affiliation)** |  |  |
| No | 3806 (58.4%) | 2043 (73.1%) |
| Yes | 1222 (18.7%) | 750 (26.9%) |
| Missing | 1490 (22.9%) |  |
| **Region of residence** |  |  |
| Northeast | 1379 (21.2%) | 772 (27.6%) |
| Midwest | 1308 (20.1%) | 756 (27.1%) |
| South | 1379 (21.2%) | 746 (26.7%) |
| West | 994 (15.3%) | 519 (18.6%) |
| Missing | 1458 (22.4%) |  |
| **Area of residence** |  |  |
| Suburban | 2697 (41.4%) | 1538 (55.1%) |
| Urban | 770 (11.8%) | 395 (14.1%) |
| Rural | 1593 (24.4%) | 860 (30.8%) |
| Missing | 1458 (22.4%) |  |
| **Mainstream media source to seek information on COVID-19** |  |  |
| Do not use mainstream media | 1804 (27.7%) | 924 (33.1%) |
| CNN | 862 (13.2%) | 473 (16.9%) |
| Fox News | 676 (10.4%) | 390 (14.0%) |
| MSNBC | 503 (7.7%) | 241 (8.6%) |
| Other local or national networks | 1288 (19.8%) | 672 (24.1%) |
| Other International networks (e.g., BBC, Al Jazeera, Sky News) | 204 (3.1%) | 93 (3.3%) |
| Missing | 1181 (18.1%) | 924 (33.1%) |
| **Moved out of the primary residence due to COVID-19** |  |  |
| No | 5148 (79.0%) | 2720 (97.4%) |
| Yes | 145 (2.2%) | 73 (2.6%) |
| Missing | 1225 (18.8%) |  |
| **COVID-19 information seeking frequency** |  |  |
| A couple of times a week | 814 (12.5%) | 422 (15.1%) |
| Multiple times a day | 2550 (39.1%) | 1377 (49.3%) |
| Never | 438 (6.7%) | 185 (6.6%) |
| Once a day | 1284 (19.7%) | 660 (23.6%) |
| Once a week | 338 (5.2%) | 149 (5.3%) |
| Missing | 1094 (16.8%) |  |
| **COVID-19 knowledge score (range 0 – 21)** |  |  |
| Mean (SD) | 16.2 (7.27) | 19.5 (1.64) |
| Median [Min, Max] | 19.0 [0, 21.0] | 20.0 [8.00, 21.0] |
| **Covered by health insurance or plan** |  |  |
| No | 357 (5.5%) | 143 (5.1%) |
| Yes | 6021 (92.4%) | 2650 (94.9%) |
| Missing | 140 (2.1%) |  |
| **Loneliness score (range 0 – 9)** |  |  |
| Mean (SD) | 3.39 (2.81) | 3.69 (2.56) |
| Median [Min, Max] | 3.00 [0, 9.00] | 4.00 [0, 9.00] |
| **Lost income due to COVID-19** |  |  |
| No | 2995 (45.9%) | 1681 (60.2%) |
| Yes | 1995 (30.6%) | 1112 (39.8%) |
| Missing | 1528 (23.4%) |  |
| **Financial concern due to COVID-19 (loss of income or investment) (range 0 – 8)** |  |  |
| Mean (SD) | 2.41 (2.13) | 2.96 (2.03) |
| Median [Min, Max] | 2.00 [0, 6.00] | 3.00 [0, 6.00] |
| **Low or very low food security** |  |  |
| No | 5338 (81.9%) | 2284 (81.8%) |
| Yes | 1180 (18.1%) | 509 (18.2%) |
| **Seek COVID-19 information from spouse/partner** |  |  |
| No | 2222 (34.1%) | 1423 (50.9%) |
| Yes | 2324 (35.7%) | 1370 (49.1%) |
| Missing | 1972 (30.3%) |  |
| **Seek COVID-19 information from other family member** |  |  |
| No | 2937 (45.1%) | 1666 (59.6%) |
| Yes | 2269 (34.8%) | 1127 (40.4%) |
| Missing | 1312 (20.1%) |  |
| **Seek COVID-19 information from friends or coworkers** |  |  |
| No | 2755 (42.3%) | 1504 (53.8%) |
| Yes | 2430 (37.3%) | 1289 (46.2%) |
| Missing | 1333 (20.5%) |  |
| **Seek COVID-19 information from religious leader** |  |  |
| No | 4584 (70.3%) | 2647 (94.8%) |
| Yes | 268 (4.1%) | 146 (5.2%) |
| Missing | 1666 (25.6%) |  |
| **Seek COVID-19 information from doctor/medical provider** |  |  |
| No | 2751 (42.2%) | 1498 (53.6%) |
| Yes | 2351 (36.1%) | 1295 (46.4%) |
| Missing | 1416 (21.7%) |  |
| **Seek COVID-19 information from TV** |  |  |
| No | 1503 (23.1%) | 823 (29.5%) |
| Yes | 3804 (58.4%) | 1970 (70.5%) |
| Missing | 1211 (18.6%) |  |
| **Seek COVID-19 information from Radio** |  |  |
| No | 2853 (43.8%) | 1514 (54.2%) |
| Yes | 2414 (37.0%) | 1279 (45.8%) |
| Missing | 1251 (19.2%) |  |
| **Seek COVID-19 information from newspaper** |  |  |
| No | 1591 (24.4%) | 848 (30.4%) |
| Yes | 3744 (57.4%) | 1945 (69.6%) |
| Missing | 1183 (18.1%) |  |
| **Seek COVID-19 information from government or other official websites (e.g., the CDC or WHO)** |  |  |
| No | 874 (13.4%) | 431 (15.4%) |
| Yes | 4512 (69.2%) | 2362 (84.6%) |
| Missing | 1132 (17.4%) |  |
| **Seek COVID-19 information from social media** |  |  |
| No | 1567 (24.0%) | 867 (31.0%) |
| Yes | 3804 (58.4%) | 1926 (69.0%) |
| Missing | 1147 (17.6%) |  |
| **Seek COVID-19 information from Google search, Wikipedia or other non-government websites** |  |  |
| No | 1739 (26.7%) | 875 (31.3%) |
| Yes | 3621 (55.6%) | 1918 (68.7%) |
| Missing | 1158 (17.8%) |  |
| **Perceived risk of getting infected with COVID-19 (range 0 – 10)** |  |  |
| Mean (SD) | 5.03 (2.36) | 5.11 (2.35) |
| Median [Min, Max] | 5.00 [0, 10.0] | 5.00 [0, 10.0] |
| Missing | 1279 (19.6%) |  |
| **Perceived severity of COVID-19 symptoms (range 0 – 10)** |  |  |
| Mean (SD) | 5.83 (2.48) | 5.68 (2.47) |
| Median [Min, Max] | 6.00 [0, 10.0] | 6.00 [0, 10.0] |
| Missing | 1279 (19.6%) |  |
| **Anxiety** |  |  |
| No | 4614 (70.8%) | 1722 (61.7%) |
| Yes | 1904 (29.2%) | 1071 (38.3%) |
| **Depression** |  |  |
| No | 5225 (80.2%) | 2085 (74.7%) |
| Yes | 1293 (19.8%) | 708 (25.3%) |
| **Have sought mental health services due to COVID-19 outbreak** |  |  |
| No | 4847 (74.4%) | 2662 (95.3%) |
| Yes | 235 (3.6%) | 131 (4.7%) |
| Missing | 1436 (22.0%) |  |


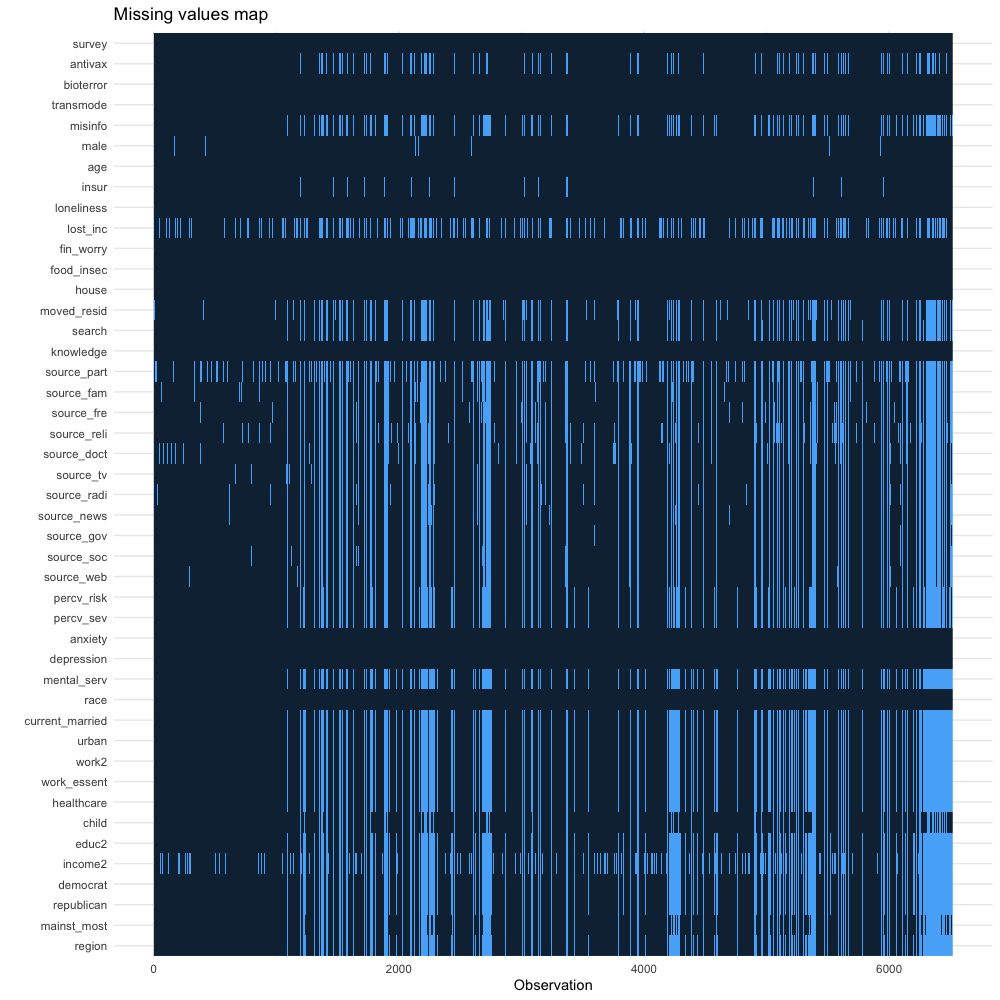


Figure S2-1. Missingness plot (light blue color representing the missing observations)


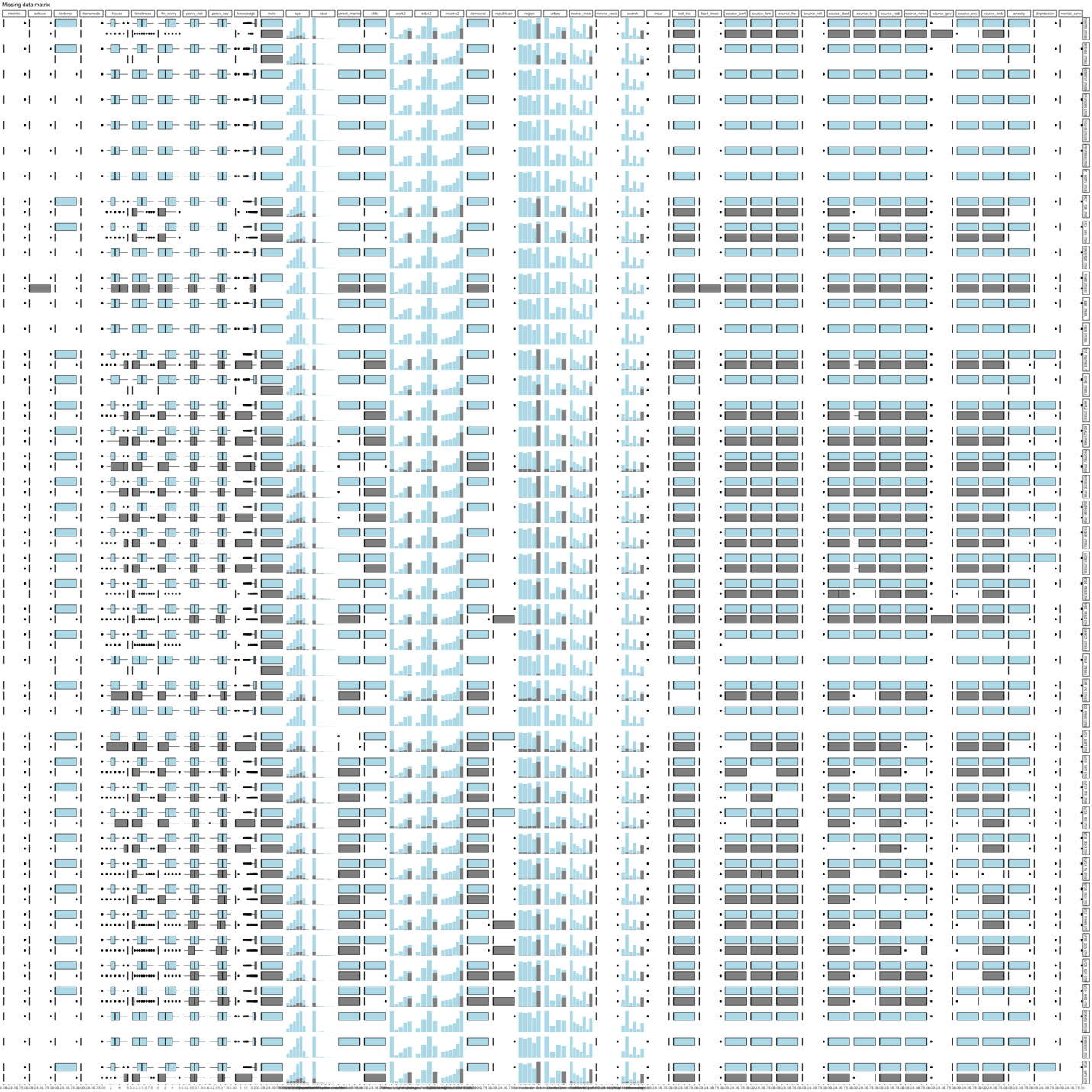


Figure S2-2. Associations between the missingness of each variable and observed data (grey color representing the missing observations)
