# Supplementary material for: What predicts people’s belief in COVID-19 misinformation? A retrospective study using a nationwide online survey among adults residing in the United States
Source: BMC Public Health. 2022 Nov 18;22:2114. doi: 10.1186/s12889-022-14431-y (PMC9673212; doi:10.1186/s12889-022-14431-y)
Supplement: Supplementary file 3 — Additional file 3. Cross-tabulation of belief in different types of misinformation. [file 12889_2022_14431_MOESM3_ESM.docx]

Supplementary Material 3: Cross-tabulation of belief in different types of misinformation


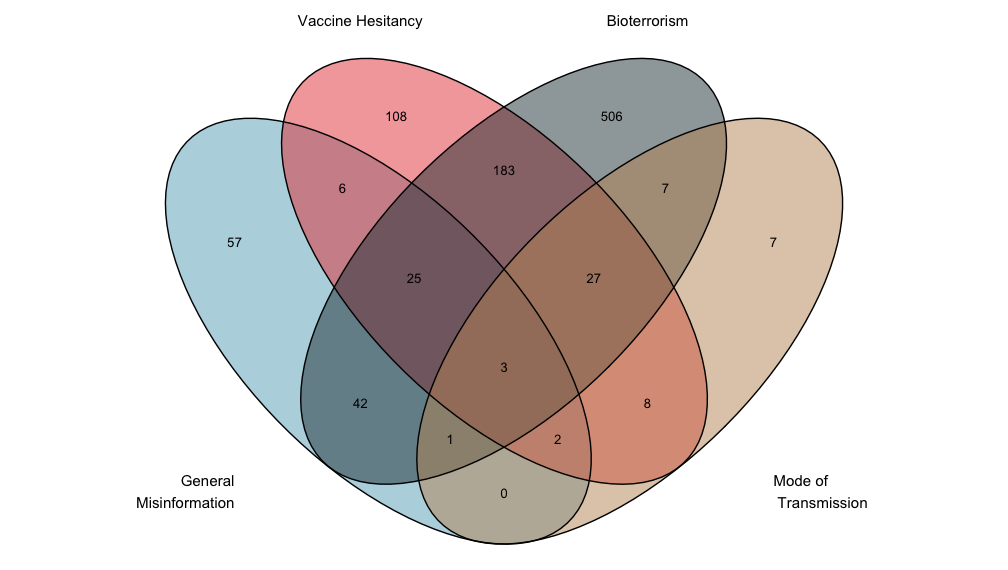


Figure S3-1. Venn diagram depicting the relationship between the belief in four different types of misinformation

Table S3-1. Pearson correlation between the belief in four different types of misinformation

|  | General  Misinformation | Vaccine hesitancy | Bioterrorism | Modes of transmission |
| --- | --- | --- | --- | --- |
| General  Misinformation |  |  |  |  |
| Vaccine hesitancy | 0.091 |  |  |  |
| Bioterrorism | 0.119 | 0.319 |  |  |
| Modes of transmission | 0.040 | 0.252 | 0.128 |  |

Table S3-2. Cross-tabulation of belief in the misinformation related to COVID-19 vaccine hesitancy and bioterrorism

|  |  | COVID-19 vaccine hesitancy | |
| --- | --- | --- | --- |
|  |  | No | Yes |
| Bioterrorism | No | 1875 | 124 |
|  | Yes | 556 | 238 |

Table S3-3. Cross-tabulation of belief in the misinformation related to COVID-19 vaccine hesitancy and mode of transmission

|  |  | COVID-19 vaccine hesitancy | |
| --- | --- | --- | --- |
|  |  | No | Yes |
| Mode of transmission | No | 2416 | 322 |
|  | Yes | 15 | 40 |

Table S3-4. Cross-tabulation of belief in the misinformation related to COVID-19 vaccine hesitancy and general misinformation

|  |  | COVID-19 vaccine hesitancy | |
| --- | --- | --- | --- |
|  |  | No | Yes |
| General misinformation | No | 2331 | 326 |
|  | Yes | 100 | 36 |

Table S3-5. Cross-tabulation of belief in the misinformation related to bioterrorism and mode of transmission

|  |  | Bioterrorism | |
| --- | --- | --- | --- |
|  |  | No | Yes |
| Mode of transmission | No | 1982 | 756 |
|  | Yes | 17 | 38 |

Table S3-6. Cross-tabulation of belief in the misinformation related to bioterrorism and general misinformation

|  |  | Bioterrorism | |
| --- | --- | --- | --- |
|  |  | No | Yes |
| General misinformation | No | 1934 | 723 |
|  | Yes | 65 | 71 |

Table S3-7. Cross-tabulation of belief in the misinformation related to mode of transmission and general misinformation

|  |  | Mode of transmission | |
| --- | --- | --- | --- |
|  |  | No | Yes |
| General misinformation | No | 2608 | 49 |
|  | Yes | 130 | 6 |
