# Supplementary material for: What predicts people’s belief in COVID-19 misinformation? A retrospective study using a nationwide online survey among adults residing in the United States
Source: BMC Public Health. 2022 Nov 18;22:2114. doi: 10.1186/s12889-022-14431-y (PMC9673212; doi:10.1186/s12889-022-14431-y)
Supplement: Supplementary file 4 — Additional file 4. Summary of the predictors for COVID-19 misinformation, adjusted odds ratios, and their 95% bootstrap confidence interval. [file 12889_2022_14431_MOESM4_ESM.docx]

Supplementary Material 4: Summary of the predictors for COVID-19 misinformation, adjusted odds ratios, and their 95% bootstrap confidence interval

Table S4-1. Summary of LASSO regression results on the general misinformation

|  | Adjusted Odds Ratio | 95 % CI Lower Bound | 95% CI Upper Bound |
| --- | --- | --- | --- |
| (Intercept) | -2.12 | -2.15 | -2.09 |
| Male | 0.19 | 0.18 | 0.20 |
| Age 40-49 Years Old | -0.25 | -0.26 | -0.23 |
| Age 80+ Years Old | 1.27 | 1.19 | 1.35 |
| Covered By Insurance | -0.37 | -0.39 | -0.35 |
| Loneliness | -0.03 | -0.03 | -0.03 |
| COVID-19 Info Search: Never | 0.18 | 0.17 | 0.20 |
| COVID-19 Info Search: Once A Week | 0.40 | 0.38 | 0.42 |
| Use Info Source: Partner | -0.20 | -0.21 | -0.19 |
| Use Info Source: Family | -0.41 | -0.42 | -0.39 |
| Use Info Source: Religious Leader | 0.40 | 0.38 | 0.42 |
| Use Info Source: TV | -0.19 | -0.21 | -0.18 |
| Use Info Source: Newspaper | -0.55 | -0.56 | -0.54 |
| Use Info Source: Government | -0.28 | -0.29 | -0.27 |
| Used Mental Health Service Due To COVID-19 | 0.99 | 0.96 | 1.01 |
| Race: Black, Non-Hispanic | 1.55 | 1.50 | 1.61 |
| Race: Native American Or American Indian | 1.51 | 1.47 | 1.56 |
| Has Child(ren) | -0.38 | -0.40 | -0.37 |
| High School Or Less | 0.77 | 0.75 | 0.78 |
| Some College / Associate's Degree | 0.21 | 0.20 | 0.23 |
| Income: 30,000 To Less Than 50,000 | 0.30 | 0.29 | 0.32 |
| Income:less Than 30,000 | 0.67 | 0.65 | 0.69 |
| Use Info Source: Do Not Use Mainstream Media | 0.07 | 0.06 | 0.09 |
| Use Info Source: Fox News | 0.23 | 0.22 | 0.25 |
| Use Info Source: Other International Networks | -0.84 | -0.89 | -0.78 |
| Use Info Source: Other Local Or National Networks | -0.28 | -0.30 | -0.27 |

Table S4-2. Summary of LASSO regression results on the misinformation related to COVID-19 anti-vaccine

|  | Adjusted Odds Ratio | 95 % CI Lower Bound | 95% CI Upper Bound |
| --- | --- | --- | --- |
| (Intercept) | 6.021 | 5.958 | 6.083 |
| Male | -0.345 | -0.355 | -0.336 |
| Age 40-49 Years Old | 0.085 | 0.075 | 0.095 |
| Age 50-59 Years Old | 0.104 | 0.096 | 0.113 |
| Age 70-79 Years Old | -0.820 | -0.845 | -0.795 |
| Age 80+ Years Old | -2.122 | -2.208 | -2.037 |
| Covered By Insurance | -0.869 | -0.885 | -0.853 |
| Loneliness | -0.082 | -0.084 | -0.081 |
| # Of People In The Household | 0.080 | 0.075 | 0.084 |
| COVID-19 Info Search: Multiple Times A Day | -0.284 | -0.294 | -0.273 |
| COVID-19 Info Search: Never | 0.622 | 0.607 | 0.637 |
| COVID-19 Info Search: Once A Day | -0.227 | -0.239 | -0.216 |
| COVID-19 Knowledge | -0.270 | -0.273 | -0.267 |
| Use Info Source: Partner | -0.111 | -0.119 | -0.103 |
| Source Doct | -0.281 | -0.291 | -0.272 |
| Use Info Source: TV | -0.124 | -0.133 | -0.116 |
| Use Info Source: Newspaper | -0.396 | -0.406 | -0.386 |
| Use Info Source: Government | -0.223 | -0.234 | -0.212 |
| Use Info Source: Social Media | 0.209 | 0.199 | 0.219 |
| Use Info Source: Web COVID-19 Info Search: | 0.224 | 0.214 | 0.233 |
| Perceived Risk | -0.066 | -0.068 | -0.064 |
| Perceived Severity | -0.203 | -0.206 | -0.201 |
| Anxiety | -0.340 | -0.351 | -0.330 |
| Used Mental Health Service Due To COVID-19 | -0.577 | -0.612 | -0.543 |
| Race: Interracial, Mixed Race, Or Other | 1.284 | 1.258 | 1.310 |
| Race: Native American Or American Indian | 0.280 | 0.240 | 0.320 |
| Currently Married | -0.671 | -0.685 | -0.658 |
| Suburban | -0.126 | -0.134 | -0.117 |
| Urban | -0.312 | -0.327 | -0.298 |
| Not Working/Unemployed | 0.194 | 0.182 | 0.207 |
| Retired | -0.216 | -0.229 | -0.204 |
| Healthcare Worker | -0.345 | -0.360 | -0.330 |
| Has Child(ren) | 0.235 | 0.224 | 0.247 |
| High School Or Less | 0.535 | 0.520 | 0.550 |
| Some College / Associate's Degree | 0.607 | 0.597 | 0.617 |
| Income: 50,000 To Less Than 75,000 | 0.496 | 0.485 | 0.507 |
| Income: Less Than 30,000 | 0.139 | 0.123 | 0.154 |
| Democrat | -0.970 | -0.984 | -0.957 |
| Republican | 0.221 | 0.211 | 0.231 |
| Use Info Source: Do Not Use Mainstream Media | 0.546 | 0.536 | 0.556 |
| Use Info Source: MSNBC | -1.172 | -1.213 | -1.131 |
| Region: South | 0.334 | 0.325 | 0.344 |
| Region: West | 0.232 | 0.221 | 0.243 |

Table S4-3. Summary of LASSO regression results on the misinformation related to bioterrorism

|  | **Adjusted Odds Ratio** | **95 % CI Lower Bound** | **95% CI Upper Bound** |
| --- | --- | --- | --- |
| (Intercept) | 5.709 | 5.661 | 5.757 |
| Male | -0.168 | -0.175 | -0.162 |
| Age 30-39 Years Old | -0.188 | -0.198 | -0.179 |
| Age 50-59 Years Old | 0.140 | 0.133 | 0.146 |
| Age 80+ Years Old | -1.634 | -1.705 | -1.564 |
| Covered By Insurance | -0.172 | -0.184 | -0.161 |
| Loneliness | -0.024 | -0.025 | -0.023 |
| Lost Inc | 0.088 | 0.082 | 0.094 |
| Food Insecurity | 0.417 | 0.409 | 0.426 |
| # Of People In The Household | 0.088 | 0.085 | 0.090 |
| COVID-19 Info Search: Multiple Times A Day | -0.062 | -0.068 | -0.056 |
| COVID-19 Info Search: Never | 0.209 | 0.198 | 0.221 |
| COVID-19 Info Search: Once A Week | 0.395 | 0.381 | 0.408 |
| COVID-19 Knowledge | -0.323 | -0.325 | -0.321 |
| Use Info Source: Partner | -0.100 | -0.106 | -0.094 |
| Use Info Source: Friends And Colleagues | 0.136 | 0.129 | 0.143 |
| Use Info Source: Religious Leader | 0.285 | 0.272 | 0.298 |
| Use Info Source: Newspaper | -0.698 | -0.705 | -0.691 |
| Use Info Source: Government | -0.258 | -0.267 | -0.250 |
| Use Info Source: Social Media | 0.346 | 0.338 | 0.353 |
| Perceived Risk | -0.016 | -0.017 | -0.015 |
| Anxiety | 0.221 | 0.213 | 0.229 |
| Race: Interracial, Mixed Race, Or Other | 0.316 | 0.296 | 0.336 |
| Race: White, Non-Hispanic | -0.394 | -0.408 | -0.380 |
| Currently Married | -0.142 | -0.151 | -0.134 |
| Suburban | -0.133 | -0.139 | -0.128 |
| Retired | 0.262 | 0.253 | 0.271 |
| Healthcare Worker | 0.267 | 0.257 | 0.277 |
| High School Or Less | 0.429 | 0.417 | 0.440 |
| Some College / Associate's Degree | 0.470 | 0.463 | 0.477 |
| Income: 50,000 To Less Than 75,000 | 0.101 | 0.094 | 0.108 |
| Democrat | -0.823 | -0.832 | -0.814 |
| Republican | 0.560 | 0.553 | 0.568 |
| Use Info Source: Do Not Use Mainstream Media | 0.390 | 0.383 | 0.398 |
| Use Info Source: Fox News | 0.722 | 0.712 | 0.733 |
| Use Info Source: MSNBC | -0.280 | -0.294 | -0.266 |
| Region: West | -0.336 | -0.345 | -0.327 |

Table S4-4. Summary of LASSO regression results on the misinformation related to the mode of Transmission

|  | Adjusted Odds Ratio | 95 % CI Lower Bound | 95% CI Upper Bound |
| --- | --- | --- | --- |
| (Intercept) | 5.182 | 5.025 | 5.338 |
| Male | 0.997 | 0.968 | 1.026 |
| Age 30-39 Years Old | 0.405 | 0.372 | 0.437 |
| Age 60-69 Years Old | -1.516 | -1.573 | -1.459 |
| Age 70-79 Years Old | -3.036 | -3.198 | -2.874 |
| Covered By Insurance | -0.788 | -0.835 | -0.740 |
| Loneliness | 0.237 | 0.231 | 0.243 |
| # Of People In The Household | 0.158 | 0.147 | 0.169 |
| Moved Residence Due To COVID-19 | 0.685 | 0.638 | 0.732 |
| COVID-19 Info Search: Never | 0.994 | 0.960 | 1.028 |
| COVID-19 Knowledge | -0.530 | -0.537 | -0.523 |
| Use Info Source: Religious Leader | -2.680 | -2.805 | -2.556 |
| Source Doct | -0.970 | -1.005 | -0.935 |
| Use Info Source: TV | -0.909 | -0.939 | -0.879 |
| Use Info Source: Newspaper | -0.727 | -0.756 | -0.699 |
| Use Info Source: Government | -0.507 | -0.533 | -0.481 |
| Use Info Source: Social Media | -0.436 | -0.465 | -0.406 |
| Use Info Source: Web COVID-19 Info Search: | -0.280 | -0.305 | -0.254 |
| Perceived Risk | -0.087 | -0.094 | -0.081 |
| Perceived Severity | -0.472 | -0.482 | -0.463 |
| Used Mental Health Service Due To COVID-19 | -3.174 | -3.324 | -3.024 |
| Race: White, Non-Hispanic | 2.255 | 2.143 | 2.366 |
| Suburban | 0.671 | 0.643 | 0.699 |
| Essential Worker | 0.163 | 0.139 | 0.187 |
| Income: 30,000 To Less Than 50,000 | -1.190 | -1.264 | -1.117 |
| Income:less Than 30,000 | -1.678 | -1.762 | -1.594 |
| Use Info Source: Do Not Use Mainstream Media | 0.253 | 0.225 | 0.281 |
| Region: South | 0.723 | 0.696 | 0.751 |
