# Supplementary material for: What predicts people’s belief in COVID-19 misinformation? A retrospective study using a nationwide online survey among adults residing in the United States
Source: BMC Public Health. 2022 Nov 18;22:2114. doi: 10.1186/s12889-022-14431-y (PMC9673212; doi:10.1186/s12889-022-14431-y)
Supplement: Supplementary file 5 — Additional file 5. [file 12889_2022_14431_MOESM5_ESM.docx]

Table S5-1. Comparison of the study sample characteristics with the United States adult population (Age 18 and older)

|  | **US adult population^1^ (N=257,064,609)** | **Total sample (N=6,518)** | **Regression Sample (N=2,793)** |
| --- | --- | --- | --- |
| **Sex** |  |  |  |
| Female | 50.9% | 3717 (57.0%) | 1610 (57.6%) |
| Male | 49.1% | 2738 (42.0%) | 1183 (42.4%) |
| Missing |  | 63 (1.0%) |  |
| **Age group** |  |  |  |
| 18-29 years old | 20.6% | 343 (5.3%) | 120 (4.3%) |
| 30-39 years old | 17.5% | 735 (11.3%) | 372 (13.3%) |
| 40-49 years old | 16.0% | 997 (15.3%) | 495 (17.7%) |
| 50-59 years old | 16.7% | 1814 (27.8%) | 863 (30.9%) |
| 60-69 years old | 15.1% | 1967 (30.2%) | 755 (27.0%) |
| 70-79 years old | 9.4% | 605 (9.3%) | 179 (6.4%) |
| 80+ years old | 4.7% | 57 (0.9%) | 9 (0.3%) |
| **Race** |  |  |  |
| White, Non-Hispanic | 64.0% | 6012 (92.2%) | 2634 (94.3%) |
| Hispanic/Latinx | 17.0% | 169 (2.6%) | 52 (1.9%) |
| Interracial, Mixed race, or Other | 2.1% | 190 (2.9%) | 63 (2.3%) |
| Asian/Pacific Islander | 3.2% | 50 (0.8%) | 15 (0.5%) |
| Black, Non-Hispanic | 12.5% | 53(0.8%) | 12 (0.4%) |
| Native American or American Indian | 1.2% | 44 (0.7%) | 17 (0.6%) |
| **Currently married** |  |  |  |
| No | 49.8% | 1475 (22.6%) | 492 (17.6%) |
| Yes | 50.2% | 3585 (55.0%) | 2301 (82.4%) |
| Missing |  | 1458 (22.4%) |  |
| **Children under 18 in the household** |  |  |  |
| No | 71.2% | 4253 (65.3%) | 1893 (67.8%) |
| Yes | 28.8% | 1477 (22.7%) | 900 (32.2%) |
| Missing |  | 788 (12.1%) |  |
| **Number of people in the household** |  |  |  |
| Mean (SD) | 2.51 (0.01) | 3.16 (1.70) | 2.84 (1.26) |
| **Employment status** |  |  |  |
| Employed | 45.4% | 2845 (43.6%) | 1832 (65.6%) |
| Student/Unpaid work | 11.7% | 280 (4.3%) | 140 (5.0%) |
| Not working/Unemployed | 21.6% | 635 (9.7%) | 325 (11.6%) |
| Retired | 21.3% | 1300 (19.9%) | 496 (17.8%) |
| Missing |  | 1458 (22.4%) |  |
| **Highest educational attainment** |  |  |  |
| High school degree / GED or less | 37.9% | 516 (7.9%) | 264 (9.5%) |
| Some college / Associate’s degree | 27.1% | 1720 (26.4%) | 944 (33.8%) |
| Bachelor’s degree or higher | 35.0% | 2792 (42.8%) | 1585 (56.7%) |
| Missing |  | 1490 (22.9%) |  |
| **Annual household income** |  |  |  |
| Less than $30,000 | 22.1% | 580 (8.9%) | 233 (8.3%) |
| $30,000 to less than $50,000 | 15.7% | 671 (10.3%) | 378 (13.5%) |
| $50,000 to less than $75,000 | 16.5% | 767 (11.8%) | 477 (17.1%) |
| $75,000 to less than $100,000 | 12.2% | 900 (13.8%) | 614 (22.0%) |
| $100,000 or more | 33.6% | 1419 (21.8%) | 1091 (39.1%) |
| Missing |  | 2181 (33.5%) |  |
| **Democrat (political affiliation)** |  |  |  |
| No | N/A | 3103 (47.6%) | 1716 (61.4%) |
| Yes | N/A | 1925 (29.5%) | 1077 (38.6%) |
| Missing |  | 1490 (22.9%) |  |
| **Republican (political affiliation)** |  |  |  |
| No | N/A | 3806 (58.4%) | 2043 (73.1%) |
| Yes | N/A | 1222 (18.7%) | 750 (26.9%) |
| Missing |  | 1490 (22.9%) |  |
| **Region of residence** |  |  |  |
| Northeast | 17.6% | 1379 (21.2%) | 772 (27.6%) |
| Midwest | 20.7% | 1308 (20.1%) | 756 (27.1%) |
| South | 38.1% | 1379 (21.2%) | 746 (26.7%) |
| West | 23.6% | 994 (15.3%) | 519 (18.6%) |
| Missing |  | 1458 (22.4%) |  |
| **Type of residence** |  |  |  |
| Suburban | N/A | 2697 (41.4%) | 1538 (55.1%) |
| Urban | N/A | 770 (11.8%) | 395 (14.1%) |
| Rural | N/A | 1593 (24.4%) | 860 (30.8%) |
| Missing | N/A | 1458 (22.4%) |  |

*N/A = Data not available

**References**

1. United States Census Bureau. Current Population Survey - Annual Social and Economic Supplements. In; 2021.
