# Supplementary material for: What predicts people’s belief in COVID-19 misinformation? A retrospective study using a nationwide online survey among adults residing in the United States
Source: BMC Public Health. 2022 Nov 18;22:2114. doi: 10.1186/s12889-022-14431-y (PMC9673212; doi:10.1186/s12889-022-14431-y)
Supplement: Supplementary file 6 — Additional file 6. [file 12889_2022_14431_MOESM6_ESM.docx]

**Supplementary Material 5: Additional analysis using the alternative outcome variables**

**Rationale**

Due to the current lack of theoretical consensus on how to define and measure different types of COVID-19 misinformation, one of the major challenges of our study was to justify the validity and reliability of our chosen outcome variables. Following the approaches used in existing studies,^10, 29, 62^ our study defined the four outcome variables using a single survey item per each type of misinformation without means to test for their validity and reliability. To choose the question to define each variable, all authors were involved in rounds of discussion to ensure theoretical validity. However, the data used for this study was not primarily designed to collected COVID-19 misinformation. Therefore, there may be some room for different interpretation on the operational definition we implemented.

In anticipation of some disagreement and questions that may rise, we repeated our analysis on 6 other outcome variables, 5 for anti-vaccine and 1 for transmission mode, that used similar but alternative survey items for their definition and included it in this section.

**Methods**

We re-coded “anti-vaccine” misinformation variable using 5 alternative survey items in place of the original question (“how likely would you be to get a Coronavirus vaccine if it was recommended by: doctor/medical provider?”). We also created one alternative “transmission mode” misinformation variable by switching one of the original questions used in our main analysis. The comparison between the definition used in main analysis and the alternative analysis is described in the table below (Table S6-1) and the Figures S6-1 and 2. Then we repeated binary logistic regression and the LASSO regularization on the new variables to identified the important predictors of belief in each misinformation defined alternatively. Nested vector bootstrapping approach was used to estimate the standard error of the LASSO coefficients.

Table S6-1. Comparison of the definition of outcome variables used in main analysis and the alternatives

| Definition used in main analysis | Alternative definition | Outcome variable name |
| --- | --- | --- |
| Anti-vaccine  1 = responded “not likely” to the question “how likely would you be to get a Coronavirus vaccine if it was recommended by: doctor/medical provider?”  0 = responded otherwise | 1 = responded “not likely” to the question “how likely would you be to get a Coronavirus vaccine if it was recommended by: friends?”  0 = responded otherwise | Anti-vaccine 2 |
|  | 1 = responded “not likely” to the question “how likely would you be to get a Coronavirus vaccine if it was recommended by: family members?”  0 = responded otherwise | Anti-vaccine 3 |
|  | 1 = responded “not likely” to the question “how likely would you be to get a Coronavirus vaccine if it was recommended by: federal government?”  0 = responded otherwise | Anti-vaccine 4 |
|  | 1 = responded “not likely” to the question “how likely would you be to get a Coronavirus vaccine if it was recommended by: religious leader(s)?”  0 = responded otherwise | Anti-vaccine 5 |
|  | 1 = responded “not likely” to the question “how likely would you be to get a Coronavirus vaccine if it was recommended by: local health authority?”  0 = responded otherwise | Anti-vaccine 6 |
|  | 1 = responded “not likely” to the question “If a Coronavirus vaccine became AVAILABLE, how confident are you that you would: Try to get the Coronavirus vaccine immediately?”  0 = responded otherwise | Anti-vaccine 7 |
| Transmission mode  1 = 1) answered “no” to “practicing social distancing” and “wearing a face mask or covering when they leave home,” and 2) responded “strongly disagree” or “disagree” to the statement “if I were ORDERED to quarantine myself due to Coronavirus, I would do so.”  0 = responded otherwise | 1 = 1) answered “no” to “practicing social distancing” and “wearing a face mask or covering when they leave home,” and 2) responded “strongly disagree” or “disagree” to the statement “if I were ASKED to self-quarantine myself due to Coronavirus, I would do so.”  0 = responded otherwise | Transmission mode 2 |


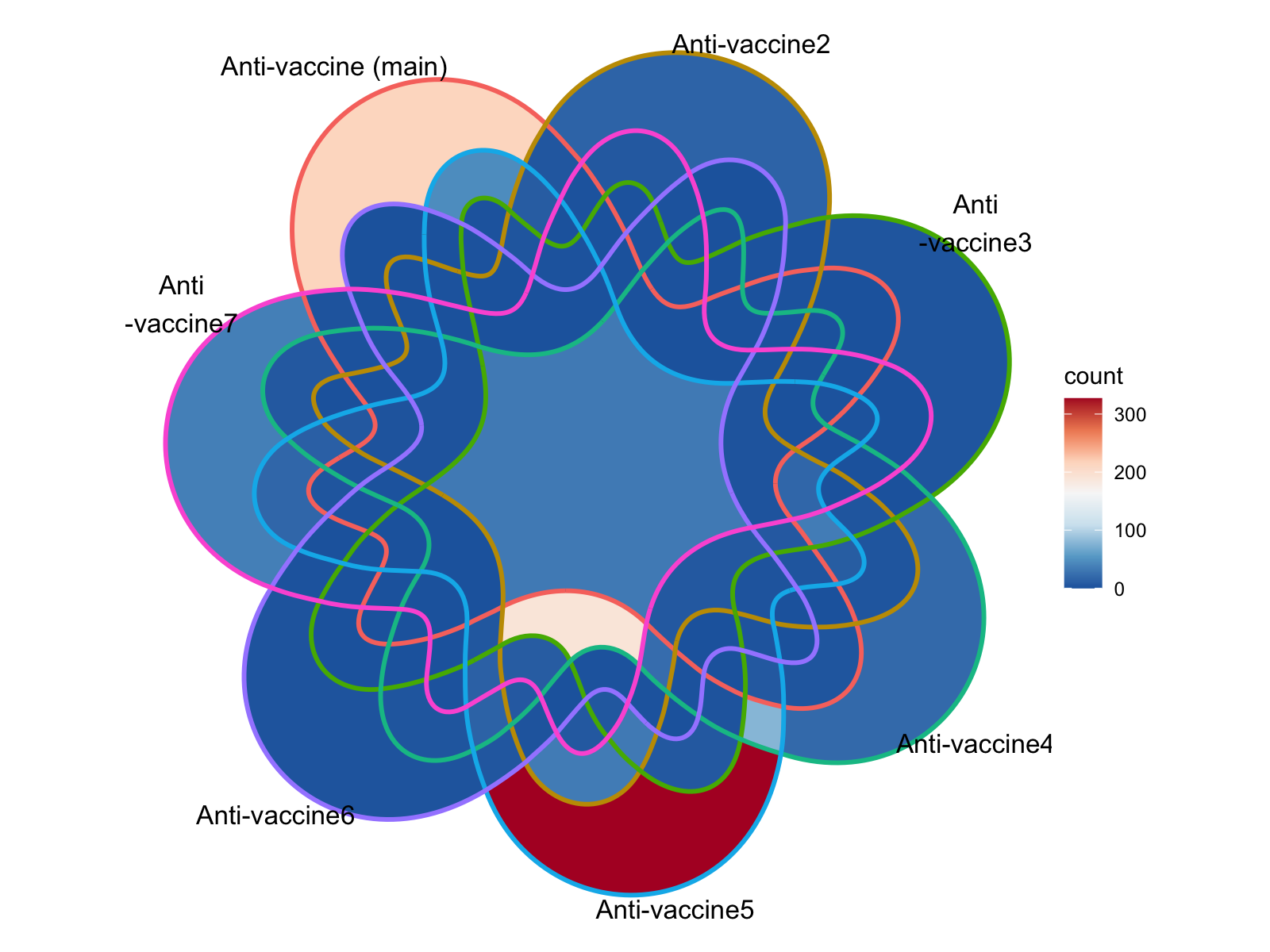


Figure S6-1. Venn diagram depicting the relationship between the belief in anti-vaccine misinformation coded in 7 different approaches


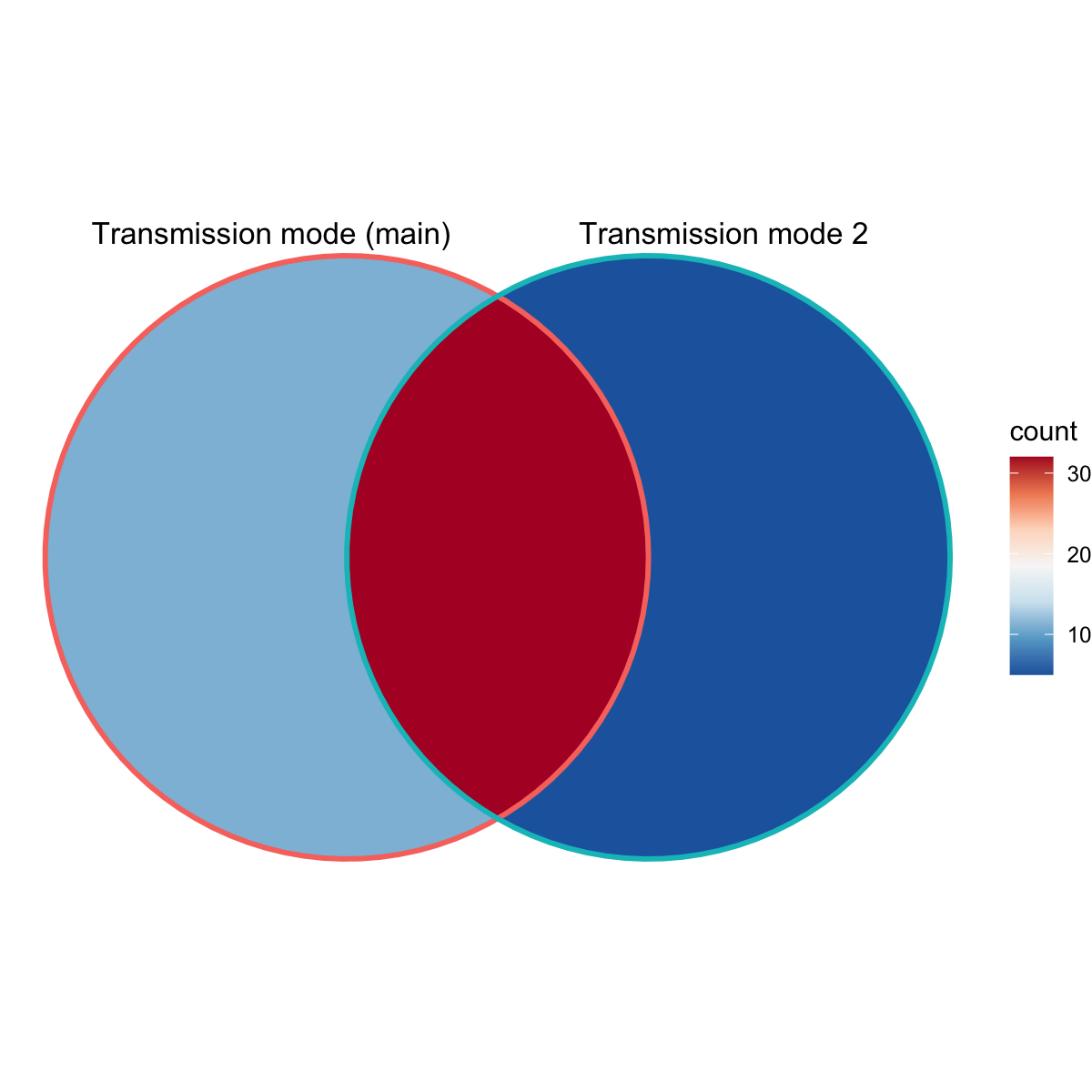


Figure S6-2. Venn diagram depicting the relationship between the belief in transmission mode-related misinformation coded in 2 different approaches

**Results**


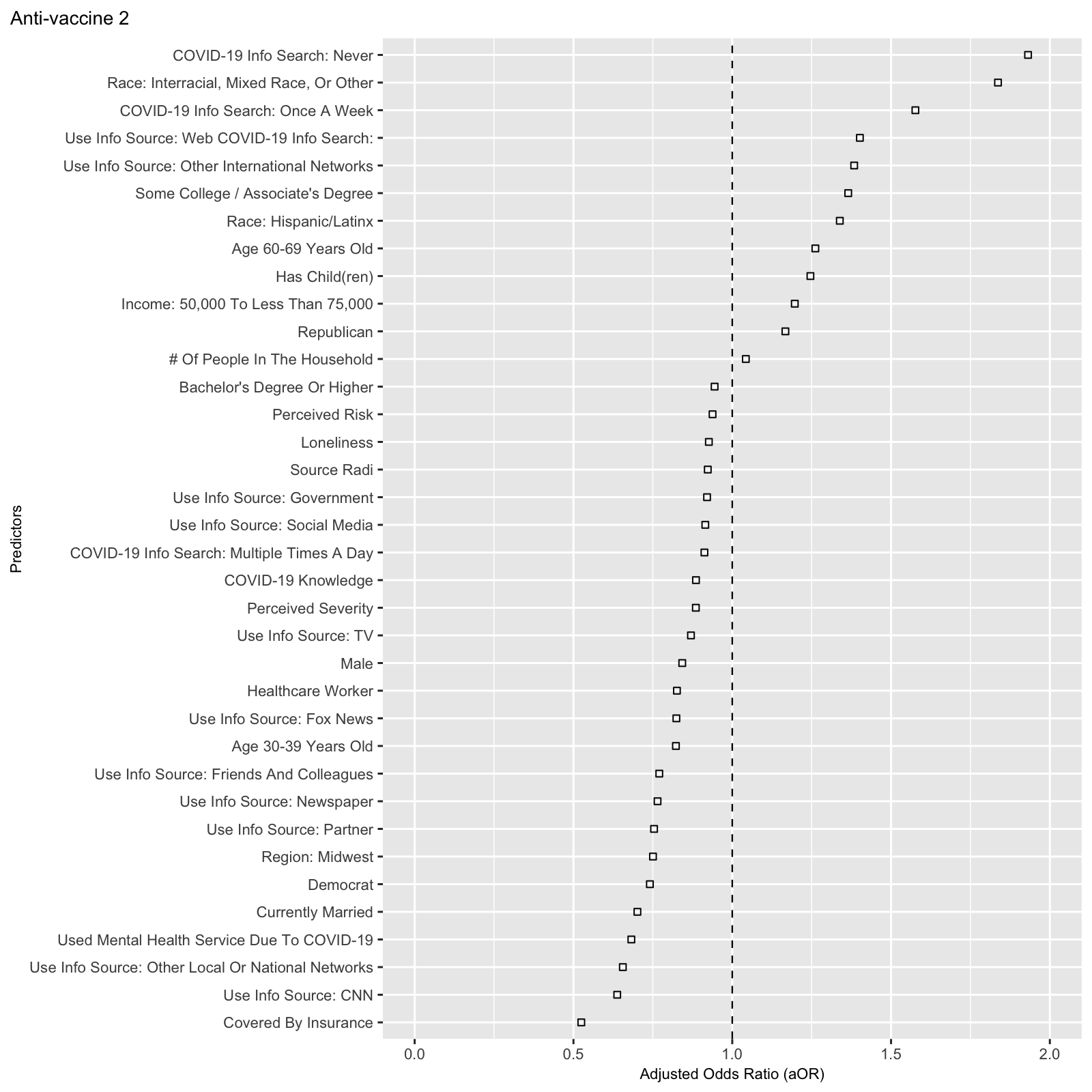


Figure S6-3. Factors associated with alternative definition of belief in anti-vaccine misinformation (see Anti-vaccine 2 in Table S6-1)

Table S6-2. Factors associated with alternative definition of belief in anti-vaccine misinformation (see Anti-vaccine 2 in Table S6-1)

|  | **Adjusted Odds Ratio** | **95 % CI Lower Bound** | **95% CI Upper Bound** |
| --- | --- | --- | --- |
| (Intercept) | 3.933 | 3.882 | 3.983 |
| Male | -0.171 | -0.179 | -0.164 |
| Age 30-39 Years Old | -0.195 | -0.206 | -0.185 |
| Age 60-69 Years Old | 0.232 | 0.223 | 0.242 |
| Covered By Insurance | -0.645 | -0.661 | -0.629 |
| Loneliness | -0.077 | -0.078 | -0.075 |
| # Of People In The Household | 0.042 | 0.039 | 0.045 |
| COVID-19 Info Search: Multiple Times A Day | -0.092 | -0.099 | -0.085 |
| COVID-19 Info Search: Never | 0.658 | 0.642 | 0.674 |
| COVID-19 Info Search: Once A Week | 0.455 | 0.441 | 0.470 |
| COVID-19 Knowledge | -0.121 | -0.124 | -0.119 |
| Use Info Source: Partner | -0.283 | -0.291 | -0.274 |
| Use Info Source: Friends And Colleagues | -0.261 | -0.269 | -0.253 |
| Use Info Source: TV | -0.139 | -0.146 | -0.132 |
| Source Radi | -0.080 | -0.087 | -0.074 |
| Use Info Source: Newspaper | -0.268 | -0.276 | -0.260 |
| Use Info Source: Government | -0.083 | -0.091 | -0.074 |
| Use Info Source: Social Media | -0.089 | -0.096 | -0.082 |
| Use Info Source: Web COVID-19 Info Search: | 0.338 | 0.329 | 0.347 |
| Perceived Risk | -0.064 | -0.066 | -0.062 |
| Perceived Severity | -0.122 | -0.124 | -0.120 |
| Used Mental Health Service Due To COVID-19 | -0.382 | -0.401 | -0.364 |
| Race: Hispanic/Latinx | 0.292 | 0.272 | 0.311 |
| Race: Interracial, Mixed Race, Or Other | 0.608 | 0.584 | 0.632 |
| Currently Married | -0.355 | -0.365 | -0.344 |
| Healthcare Worker | -0.191 | -0.201 | -0.181 |
| Has Child(ren) | 0.220 | 0.210 | 0.230 |
| Some College / Associate's Degree | 0.311 | 0.299 | 0.323 |
| Bachelor's Degree Or Higher | -0.057 | -0.067 | -0.047 |
| Income: 50,000 To Less Than 75,000 | 0.180 | 0.171 | 0.189 |
| Democrat | -0.300 | -0.310 | -0.291 |
| Republican | 0.155 | 0.146 | 0.163 |
| Use Info Source: CNN | -0.450 | -0.463 | -0.437 |
| Use Info Source: Fox News | -0.194 | -0.204 | -0.183 |
| Use Info Source: Other Local Or National Networks | -0.422 | -0.433 | -0.412 |
| Use Info Source: Other International Networks | 0.325 | 0.307 | 0.343 |
| Region: Midwest | -0.287 | -0.295 | -0.279 |


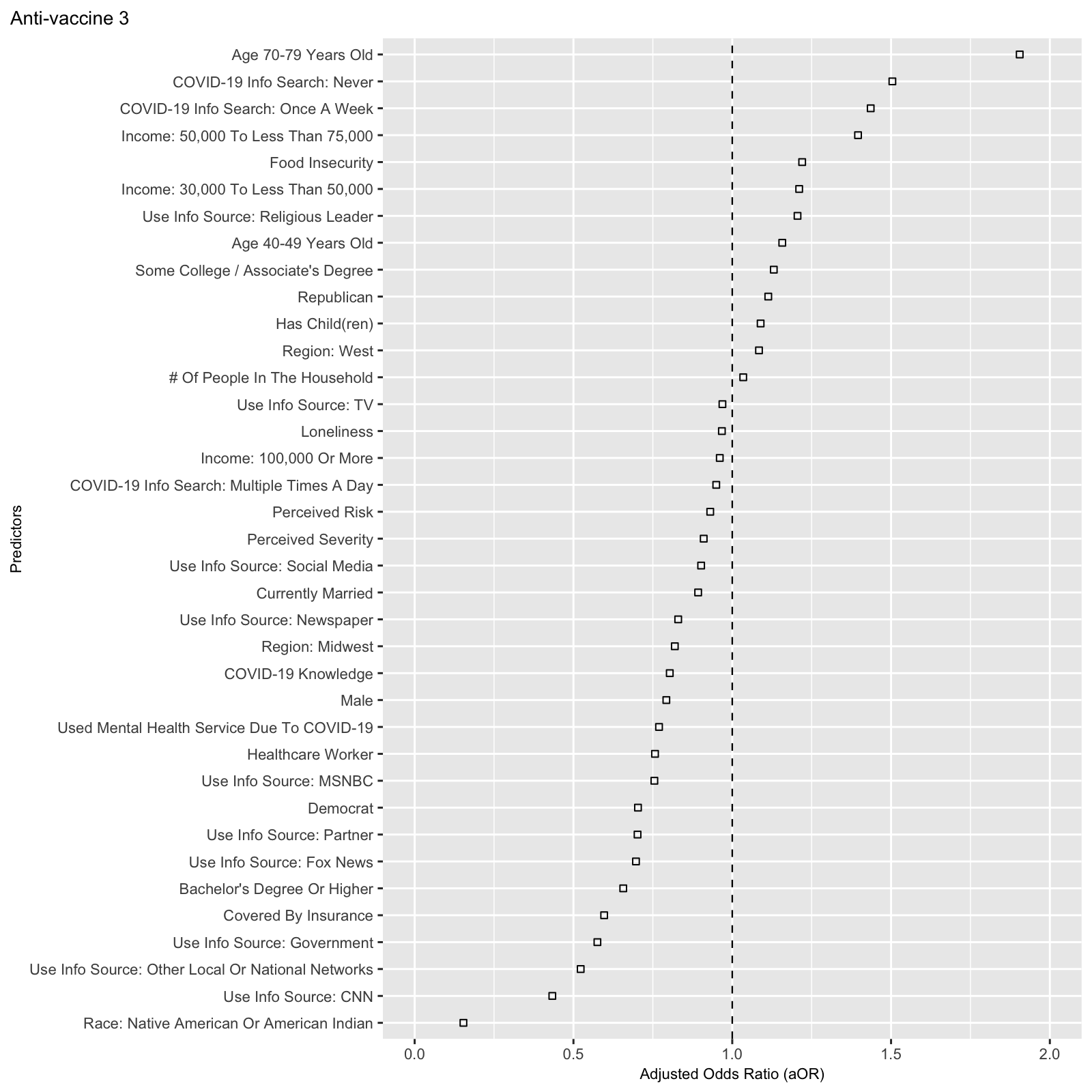


Figure S6-4. Factors associated with alternative definition of belief in anti-vaccine misinformation (see Anti-vaccine 3 in Table S6-1)

Table S6-3. Factors associated with alternative definition of belief in anti-vaccine misinformation (see Anti-vaccine 3 in Table S6-1)

|  | **Adjusted Odds Ratio** | **95 % CI Lower Bound** | **95% CI Upper Bound** |
| --- | --- | --- | --- |
| (Intercept) | 3.825 | 3.773 | 3.878 |
| Male | -0.284 | -0.293 | -0.275 |
| Age 60-69 Years Old | 0.226 | 0.215 | 0.237 |
| Covered By Insurance | -0.660 | -0.679 | -0.641 |
| Loneliness | -0.055 | -0.056 | -0.053 |
| Lost Inc | 0.215 | 0.207 | 0.223 |
| # Of People In The Household | 0.068 | 0.065 | 0.072 |
| COVID-19 Info Search: Never | 0.651 | 0.636 | 0.666 |
| COVID-19 Info Search: Once A Week | 0.349 | 0.334 | 0.363 |
| COVID-19 Knowledge | -0.118 | -0.120 | -0.115 |
| Use Info Source: Partner | -0.219 | -0.228 | -0.210 |
| Use Info Source: Family | -0.164 | -0.173 | -0.154 |
| Use Info Source: Friends And Colleagues | -0.202 | -0.210 | -0.193 |
| Use Info Source: TV | -0.282 | -0.291 | -0.273 |
| Source Radi | -0.087 | -0.095 | -0.080 |
| Use Info Source: Newspaper | -0.240 | -0.250 | -0.231 |
| Use Info Source: Government | -0.194 | -0.204 | -0.183 |
| Perceived Risk | -0.029 | -0.031 | -0.027 |
| Perceived Severity | -0.180 | -0.182 | -0.178 |
| Race: Hispanic/Latinx | 0.417 | 0.391 | 0.444 |
| Race: Interracial, Mixed Race, Or Other | 0.871 | 0.846 | 0.896 |
| Currently Married | -0.373 | -0.384 | -0.361 |
| Healthcare Worker | -0.163 | -0.174 | -0.153 |
| Has Child(ren) | 0.134 | 0.124 | 0.143 |
| Some College / Associate's Degree | 0.203 | 0.190 | 0.216 |
| Bachelor's Degree Or Higher | -0.299 | -0.312 | -0.286 |
| Income: 50,000 To Less Than 75,000 | 0.327 | 0.316 | 0.337 |
| Income: 75,000 To Less Than 100,000 | -0.118 | -0.127 | -0.109 |
| Democrat | -0.446 | -0.456 | -0.435 |
| Republican | 0.236 | 0.226 | 0.246 |
| Use Info Source: CNN | -0.516 | -0.531 | -0.502 |
| Use Info Source: Fox News | -0.298 | -0.311 | -0.285 |
| Use Info Source: Other Local Or National Networks | -0.339 | -0.351 | -0.328 |
| Region: Midwest | -0.155 | -0.165 | -0.146 |
| Region: South | 0.212 | 0.202 | 0.221 |


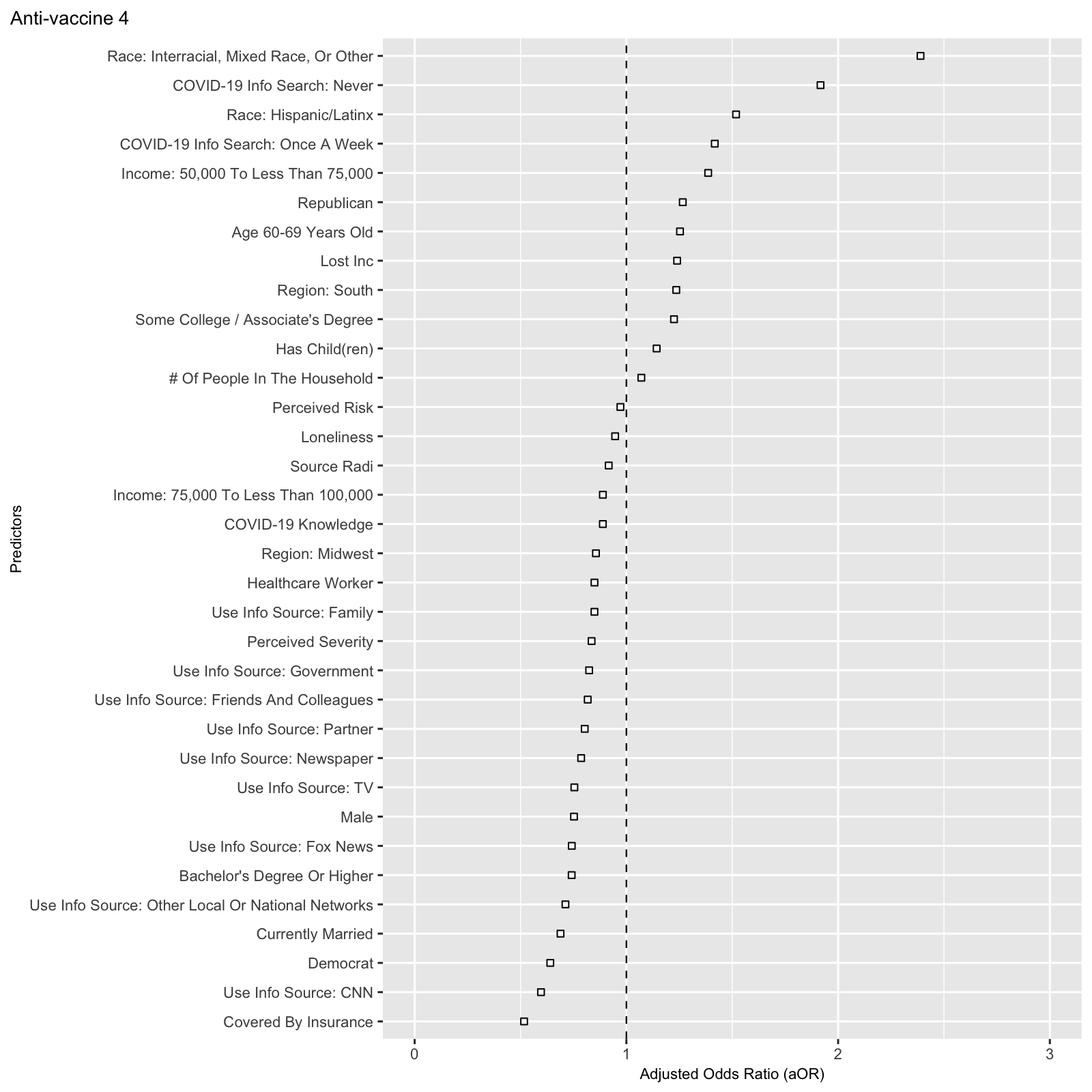


Figure S6-5. Factors associated with alternative definition of belief in anti-vaccine misinformation (see Anti-vaccine 4 in Table S6-1)

Table S6-4. Factors associated with alternative definition of belief in anti-vaccine misinformation (see Anti-vaccine 4 in Table S6-1)

|  | **Adjusted Odds Ratio** | **95 % CI Lower Bound** | **95% CI Upper Bound** |
| --- | --- | --- | --- |
| (Intercept) | 6.231 | 6.177 | 6.286 |
| Male | -0.233 | -0.241 | -0.225 |
| Age 40-49 Years Old | 0.146 | 0.138 | 0.155 |
| Age 70-79 Years Old | 0.645 | 0.620 | 0.669 |
| Covered By Insurance | -0.516 | -0.534 | -0.499 |
| Loneliness | -0.033 | -0.035 | -0.032 |
| Food Insecurity | 0.199 | 0.189 | 0.209 |
| # Of People In The Household | 0.034 | 0.031 | 0.037 |
| COVID-19 Info Search: Multiple Times A Day | -0.052 | -0.058 | -0.045 |
| COVID-19 Info Search: Never | 0.408 | 0.393 | 0.423 |
| COVID-19 Info Search: Once A Week | 0.362 | 0.347 | 0.377 |
| COVID-19 Knowledge | -0.219 | -0.222 | -0.217 |
| Use Info Source: Partner | -0.354 | -0.362 | -0.346 |
| Use Info Source: Religious Leader | 0.187 | 0.173 | 0.201 |
| Use Info Source: TV | -0.031 | -0.038 | -0.025 |
| Use Info Source: Newspaper | -0.187 | -0.195 | -0.179 |
| Use Info Source: Government | -0.553 | -0.563 | -0.542 |
| Use Info Source: Social Media | -0.103 | -0.110 | -0.096 |
| Perceived Risk | -0.072 | -0.074 | -0.070 |
| Perceived Severity | -0.094 | -0.096 | -0.092 |
| Used Mental Health Service Due To COVID-19 | -0.262 | -0.278 | -0.246 |
| Race: Native American Or American Indian | -1.874 | -1.945 | -1.803 |
| Currently Married | -0.114 | -0.122 | -0.105 |
| Healthcare Worker | -0.279 | -0.289 | -0.268 |
| Has Child(ren) | 0.086 | 0.078 | 0.093 |
| Some College / Associate's Degree | 0.123 | 0.113 | 0.133 |
| Bachelor's Degree Or Higher | -0.420 | -0.432 | -0.409 |
| Income: 30,000 To Less Than 50,000 | 0.191 | 0.180 | 0.202 |
| Income: 50,000 To Less Than 75,000 | 0.334 | 0.323 | 0.344 |
| Income: 100,000 Or More | -0.040 | -0.047 | -0.033 |
| Democrat | -0.352 | -0.361 | -0.343 |
| Republican | 0.107 | 0.100 | 0.115 |
| Use Info Source: CNN | -0.836 | -0.849 | -0.823 |
| Use Info Source: Fox News | -0.361 | -0.373 | -0.349 |
| Use Info Source: MSNBC | -0.281 | -0.297 | -0.266 |
| Use Info Source: Other Local Or National Networks | -0.649 | -0.659 | -0.638 |
| Region: Midwest | -0.199 | -0.208 | -0.191 |
| Region: West | 0.081 | 0.073 | 0.089 |


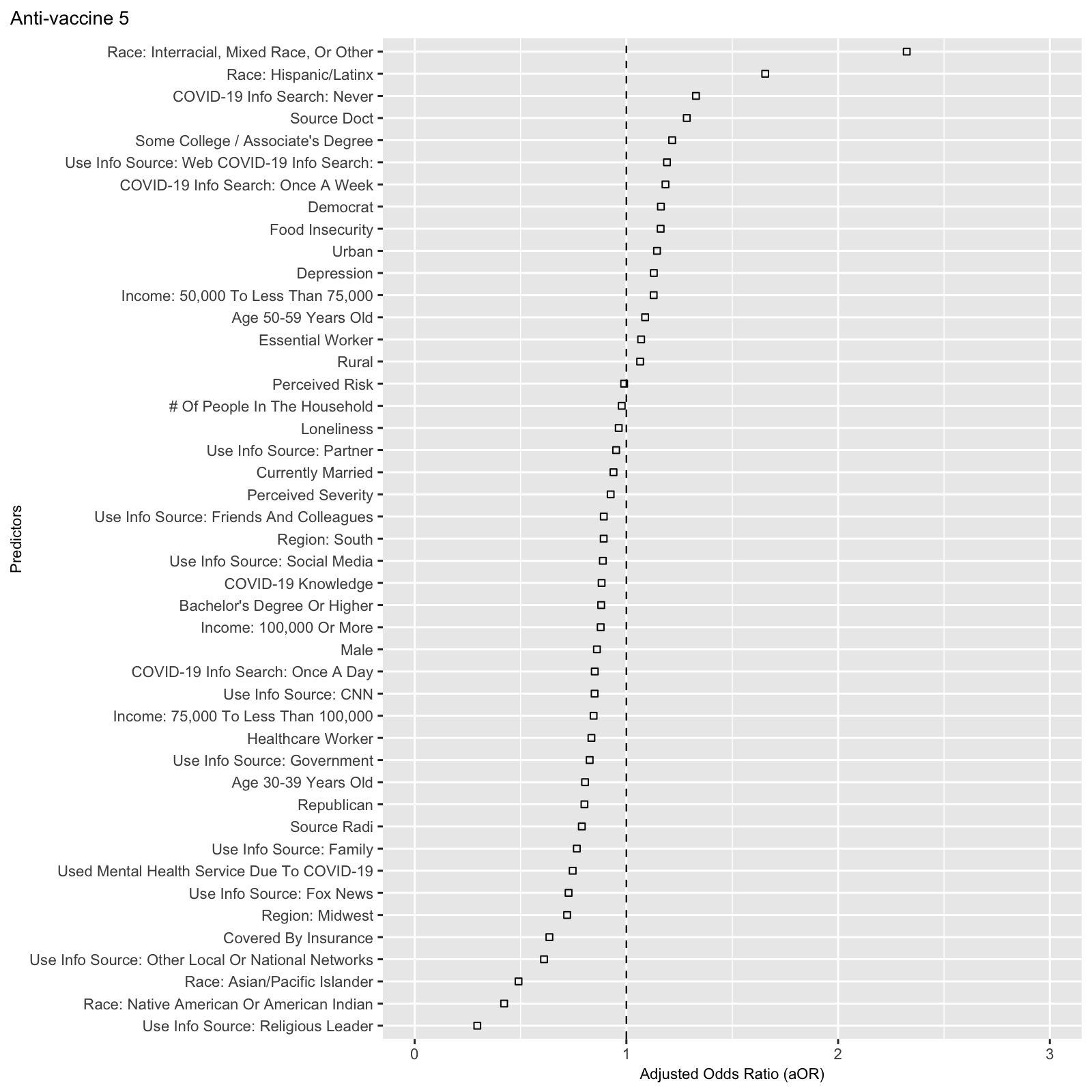


Figure S6-6. Factors associated with alternative definition of belief in anti-vaccine misinformation (see Anti-vaccine 5 in Table S6-1)

Table S6-5. Factors associated with alternative definition of belief in anti-vaccine misinformation (see Anti-vaccine 5 in Table S6-1)

|  | **Adjusted Odds Ratio** | **95 % CI Lower Bound** | **95% CI Upper Bound** |
| --- | --- | --- | --- |
| (Intercept) | 4.508 | 4.461 | 4.555 |
| Male | -0.150 | -0.156 | -0.143 |
| Age 30-39 Years Old | -0.217 | -0.226 | -0.208 |
| Age 50-59 Years Old | 0.085 | 0.079 | 0.091 |
| Covered By Insurance | -0.452 | -0.469 | -0.435 |
| Loneliness | -0.037 | -0.038 | -0.036 |
| Food Insecurity | 0.150 | 0.142 | 0.158 |
| # Of People In The Household | -0.022 | -0.024 | -0.020 |
| COVID-19 Info Search: Never | 0.284 | 0.270 | 0.298 |
| COVID-19 Info Search: Once A Day | -0.162 | -0.169 | -0.155 |
| COVID-19 Info Search: Once A Week | 0.169 | 0.157 | 0.182 |
| COVID-19 Knowledge | -0.124 | -0.127 | -0.122 |
| Use Info Source: Partner | -0.050 | -0.055 | -0.044 |
| Use Info Source: Family | -0.267 | -0.274 | -0.259 |
| Use Info Source: Friends And Colleagues | -0.113 | -0.120 | -0.106 |
| Use Info Source: Religious Leader | -1.218 | -1.235 | -1.202 |
| Source Doct | 0.251 | 0.244 | 0.257 |
| Source Radi | -0.236 | -0.243 | -0.230 |
| Use Info Source: Government | -0.191 | -0.199 | -0.182 |
| Use Info Source: Social Media | -0.118 | -0.125 | -0.111 |
| Use Info Source: Web COVID-19 Info Search: | 0.175 | 0.168 | 0.183 |
| Perceived Risk | -0.011 | -0.012 | -0.009 |
| Perceived Severity | -0.077 | -0.079 | -0.076 |
| Depression | 0.122 | 0.115 | 0.129 |
| Used Mental Health Service Due To COVID-19 | -0.293 | -0.307 | -0.278 |
| Race: Hispanic/Latinx | 0.504 | 0.481 | 0.528 |
| Race: Interracial, Mixed Race, Or Other | 0.843 | 0.821 | 0.866 |
| Race: Asian/Pacific Islander | -0.712 | -0.753 | -0.672 |
| Race: Native American Or American Indian | -0.861 | -0.916 | -0.806 |
| Currently Married | -0.063 | -0.071 | -0.055 |
| Urban | 0.135 | 0.127 | 0.144 |
| Rural | 0.063 | 0.057 | 0.069 |
| Essential Worker | 0.067 | 0.061 | 0.073 |
| Healthcare Worker | -0.181 | -0.189 | -0.172 |
| Some College / Associate's Degree | 0.196 | 0.186 | 0.205 |
| Bachelor's Degree Or Higher | -0.127 | -0.135 | -0.118 |
| Income: 50,000 To Less Than 75,000 | 0.121 | 0.113 | 0.130 |
| Income: 75,000 To Less Than 100,000 | -0.168 | -0.177 | -0.159 |
| Income: 100,000 Or More | -0.129 | -0.137 | -0.122 |
| Democrat | 0.151 | 0.144 | 0.158 |
| Republican | -0.221 | -0.229 | -0.213 |
| Use Info Source: CNN | -0.163 | -0.171 | -0.154 |
| Use Info Source: Fox News | -0.319 | -0.329 | -0.309 |
| Use Info Source: Other Local Or National Networks | -0.493 | -0.501 | -0.484 |
| Region: Midwest | -0.328 | -0.336 | -0.321 |
| Region: South | -0.114 | -0.120 | -0.107 |


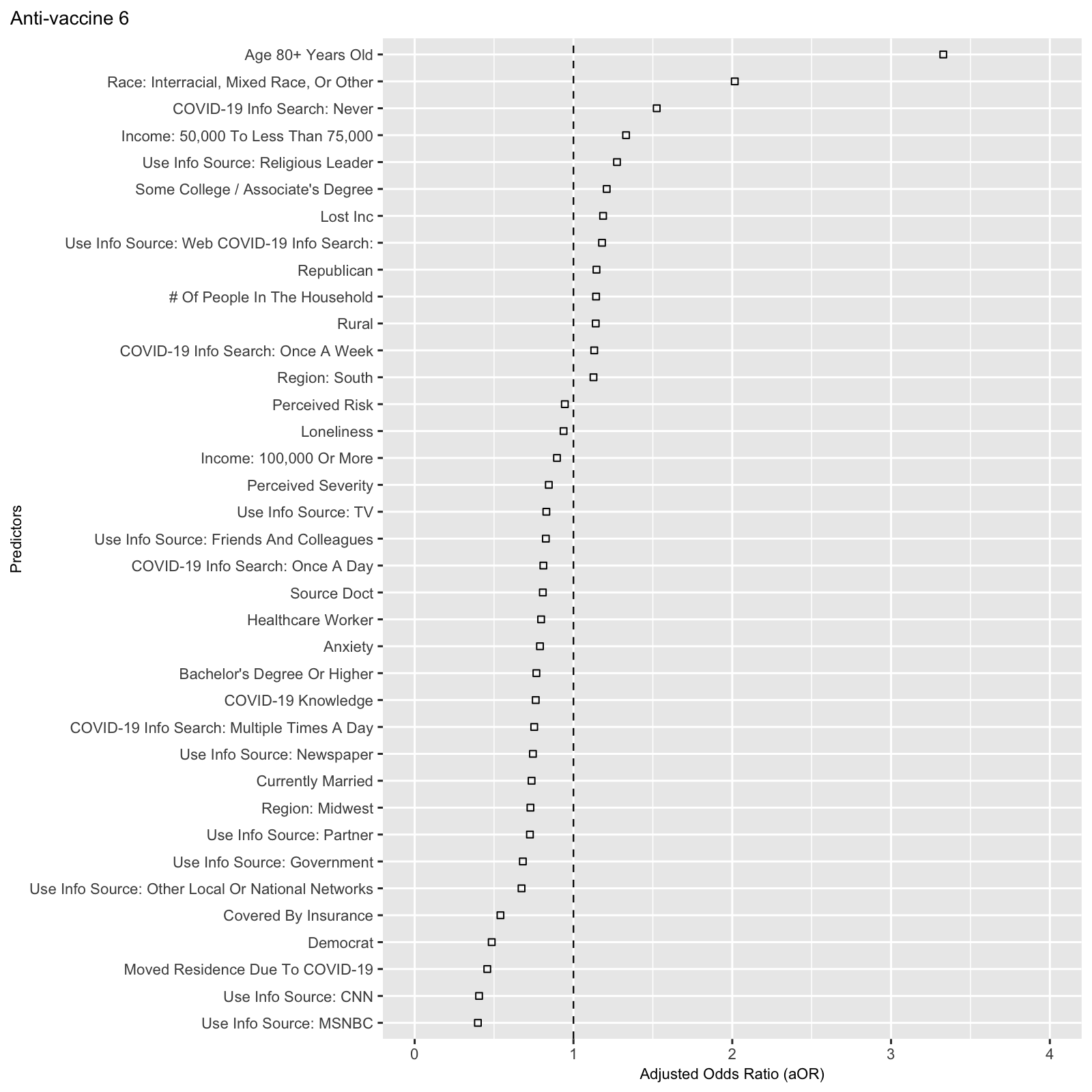


Figure S6-7. Factors associated with alternative definition of belief in anti-vaccine misinformation (see Anti-vaccine 6 in Table S6-1)

Table S6-6. Factors associated with alternative definition of belief in anti-vaccine misinformation (see Anti-vaccine 6 in Table S6-1)

|  | **Adjusted Odds Ratio** | **95 % CI Lower Bound** | **95% CI Upper Bound** |
| --- | --- | --- | --- |
| (Intercept) | 6.796 | 6.738 | 6.855 |
| Age 80+ Years Old | 1.203 | 1.126 | 1.279 |
| Covered By Insurance | -0.616 | -0.636 | -0.596 |
| Loneliness | -0.064 | -0.066 | -0.062 |
| Lost Inc | 0.171 | 0.162 | 0.180 |
| # Of People In The Household | 0.133 | 0.129 | 0.137 |
| Moved Residence Due To COVID-19 | -0.782 | -0.816 | -0.749 |
| COVID-19 Info Search: Multiple Times A Day | -0.284 | -0.295 | -0.272 |
| COVID-19 Info Search: Never | 0.422 | 0.404 | 0.439 |
| COVID-19 Info Search: Once A Day | -0.210 | -0.222 | -0.198 |
| COVID-19 Info Search: Once A Week | 0.123 | 0.107 | 0.139 |
| COVID-19 Knowledge | -0.271 | -0.274 | -0.268 |
| Use Info Source: Partner | -0.320 | -0.331 | -0.309 |
| Use Info Source: Friends And Colleagues | -0.191 | -0.200 | -0.182 |
| Use Info Source: Religious Leader | 0.242 | 0.225 | 0.260 |
| Source Doct | -0.214 | -0.224 | -0.205 |
| Use Info Source: TV | -0.187 | -0.196 | -0.178 |
| Use Info Source: Newspaper | -0.295 | -0.305 | -0.284 |
| Use Info Source: Government | -0.384 | -0.395 | -0.372 |
| Use Info Source: Web COVID-19 Info Search: | 0.165 | 0.156 | 0.175 |
| Perceived Risk | -0.056 | -0.058 | -0.054 |
| Perceived Severity | -0.169 | -0.171 | -0.166 |
| Anxiety | -0.237 | -0.247 | -0.227 |
| Race: Interracial, Mixed Race, Or Other | 0.701 | 0.672 | 0.730 |
| Currently Married | -0.306 | -0.320 | -0.293 |
| Rural | 0.131 | 0.123 | 0.140 |
| Healthcare Worker | -0.228 | -0.240 | -0.215 |
| Some College / Associate's Degree | 0.190 | 0.178 | 0.202 |
| Bachelor's Degree Or Higher | -0.266 | -0.279 | -0.253 |
| Income: 50,000 To Less Than 75,000 | 0.286 | 0.274 | 0.298 |
| Income: 100,000 Or More | -0.110 | -0.119 | -0.101 |
| Democrat | -0.723 | -0.736 | -0.710 |
| Republican | 0.135 | 0.127 | 0.144 |
| Use Info Source: CNN | -0.902 | -0.921 | -0.883 |
| Use Info Source: MSNBC | -0.921 | -0.951 | -0.891 |
| Use Info Source: Other Local Or National Networks | -0.396 | -0.408 | -0.384 |
| Region: Midwest | -0.316 | -0.327 | -0.305 |
| Region: South | 0.119 | 0.110 | 0.128 |


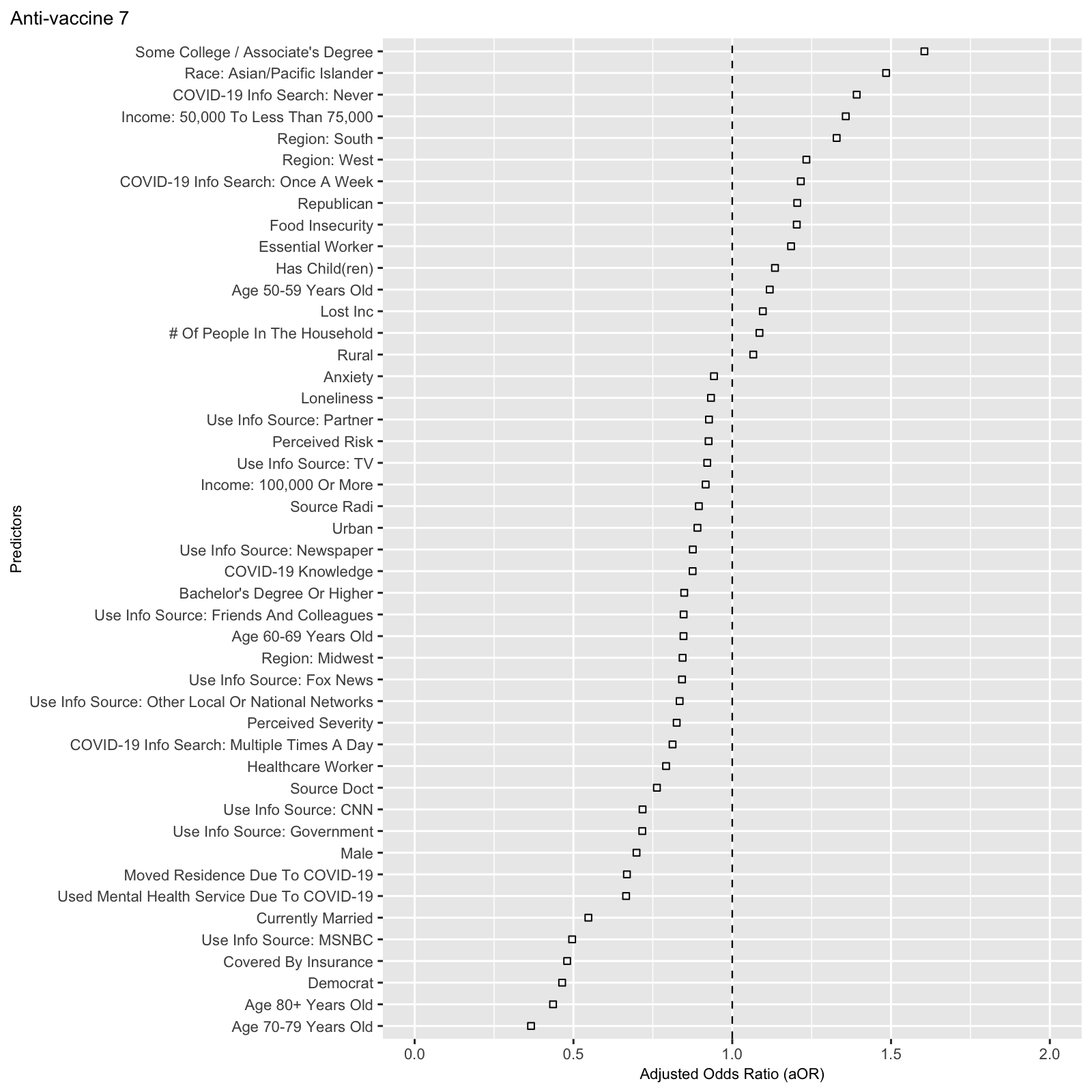


Figure S6-8. Factors associated with alternative definition of belief in anti-vaccine misinformation (see Anti-vaccine 7 in Table S6-1)

Table S6-7. Factors associated with alternative definition of belief in anti-vaccine misinformation (see Anti-vaccine 7 in Table S6-1)

|  | **Adjusted Odds Ratio** | **95 % CI Lower Bound** | **95% CI Upper Bound** |
| --- | --- | --- | --- |
| (Intercept) | 4.672 | 4.614 | 4.729 |
| Male | -0.359 | -0.368 | -0.350 |
| Age 50-59 Years Old | 0.112 | 0.103 | 0.120 |
| Age 60-69 Years Old | -0.167 | -0.178 | -0.156 |
| Age 70-79 Years Old | -1.004 | -1.041 | -0.967 |
| Age 80+ Years Old | -0.831 | -0.883 | -0.778 |
| Covered By Insurance | -0.734 | -0.753 | -0.714 |
| Loneliness | -0.069 | -0.071 | -0.067 |
| Lost Inc | 0.092 | 0.084 | 0.099 |
| Food Insecurity | 0.185 | 0.175 | 0.195 |
| # Of People In The Household | 0.082 | 0.078 | 0.086 |
| Moved Residence Due To COVID-19 | -0.403 | -0.429 | -0.377 |
| COVID-19 Info Search: Multiple Times A Day | -0.208 | -0.217 | -0.200 |
| COVID-19 Info Search: Never | 0.331 | 0.316 | 0.345 |
| COVID-19 Info Search: Once A Week | 0.195 | 0.182 | 0.209 |
| COVID-19 Knowledge | -0.133 | -0.136 | -0.131 |
| Use Info Source: Partner | -0.076 | -0.084 | -0.068 |
| Use Info Source: Friends And Colleagues | -0.166 | -0.175 | -0.158 |
| Source Doct | -0.271 | -0.279 | -0.262 |
| Use Info Source: TV | -0.082 | -0.089 | -0.075 |
| Source Radi | -0.111 | -0.119 | -0.104 |
| Use Info Source: Newspaper | -0.133 | -0.141 | -0.124 |
| Use Info Source: Government | -0.333 | -0.344 | -0.322 |
| Perceived Risk | -0.077 | -0.079 | -0.075 |
| Perceived Severity | -0.192 | -0.194 | -0.190 |
| Anxiety | -0.059 | -0.067 | -0.052 |
| Used Mental Health Service Due To COVID-19 | -0.407 | -0.431 | -0.383 |
| Race: Asian/Pacific Islander | 0.395 | 0.351 | 0.439 |
| Currently Married | -0.603 | -0.616 | -0.591 |
| Urban | -0.116 | -0.127 | -0.106 |
| Rural | 0.064 | 0.057 | 0.071 |
| Essential Worker | 0.170 | 0.161 | 0.179 |
| Healthcare Worker | -0.234 | -0.245 | -0.222 |
| Has Child(ren) | 0.126 | 0.117 | 0.136 |
| Some College / Associate's Degree | 0.473 | 0.459 | 0.487 |
| Bachelor's Degree Or Higher | -0.164 | -0.176 | -0.152 |
| Income: 50,000 To Less Than 75,000 | 0.306 | 0.295 | 0.316 |
| Income: 100,000 Or More | -0.087 | -0.095 | -0.080 |
| Democrat | -0.767 | -0.778 | -0.756 |
| Republican | 0.186 | 0.177 | 0.195 |
| Use Info Source: CNN | -0.332 | -0.345 | -0.319 |
| Use Info Source: Fox News | -0.172 | -0.183 | -0.161 |
| Use Info Source: MSNBC | -0.701 | -0.726 | -0.677 |
| Use Info Source: Other Local Or National Networks | -0.181 | -0.191 | -0.171 |
| Region: Midwest | -0.170 | -0.180 | -0.160 |
| Region: South | 0.284 | 0.274 | 0.294 |
| Region: West | 0.210 | 0.198 | 0.221 |


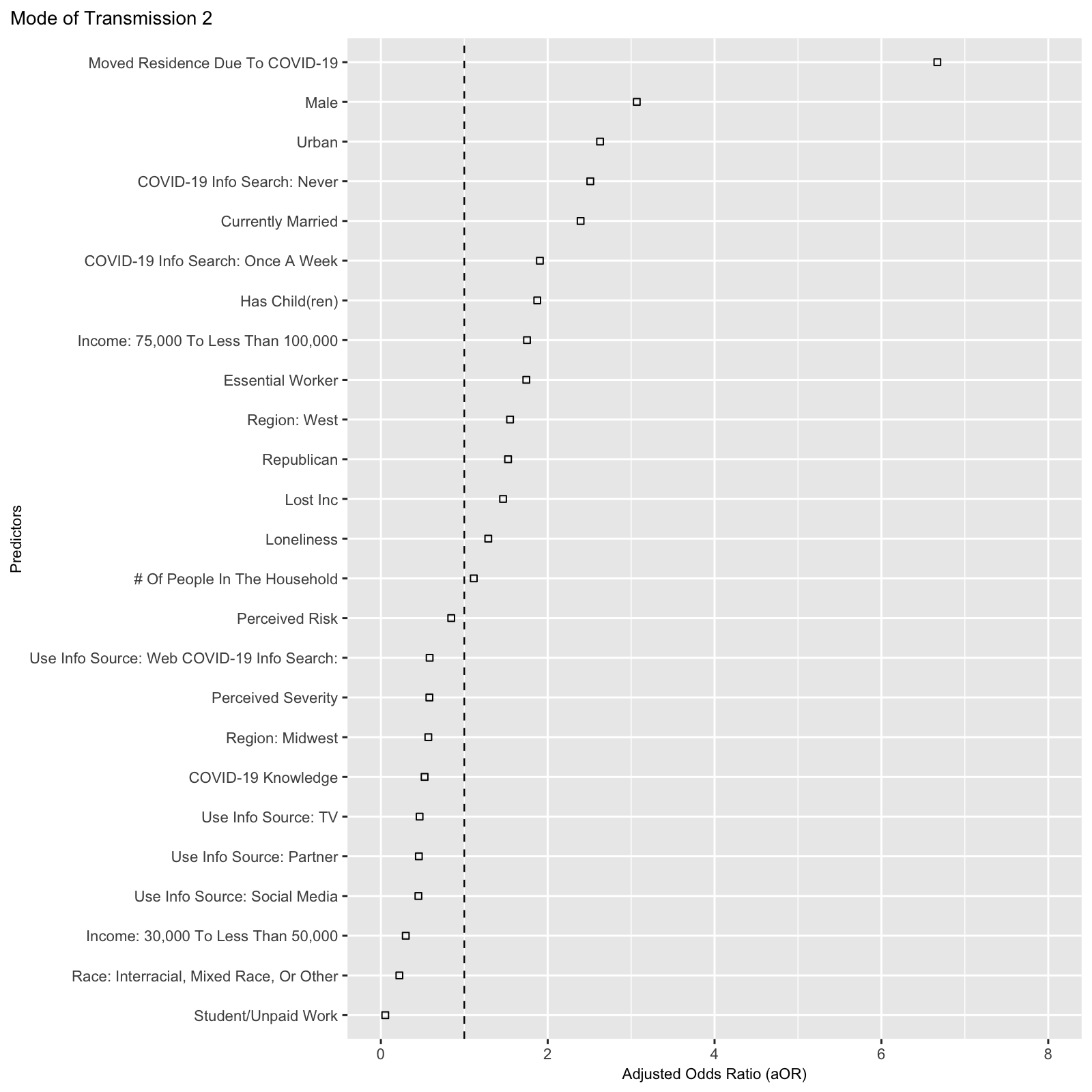


Figure S6-9. Factors associated with alternative definition of belief in transmission mode misinformation (see Transmission mode 2 in Table S6-1)

Table S6-8. Factors associated with alternative definition of belief in transmission mode misinformation (see Transmission mode 2 in Table S6-1)

|  | **Adjusted Odds Ratio** | **95 % CI Lower Bound** | **95% CI Upper Bound** |
| --- | --- | --- | --- |
| (Intercept) | 6.841 | 6.452 | 7.230 |
| Male | 1.121 | 0.998 | 1.243 |
| Loneliness | 0.253 | 0.232 | 0.274 |
| Lost Inc | 0.382 | 0.336 | 0.427 |
| # Of People In The Household | 0.108 | 0.072 | 0.143 |
| Moved Residence Due To COVID-19 | 1.898 | 1.749 | 2.046 |
| COVID-19 Info Search: Never | 0.921 | 0.807 | 1.034 |
| COVID-19 Info Search: Once A Week | 0.645 | 0.563 | 0.727 |
| COVID-19 Knowledge | -0.646 | -0.677 | -0.615 |
| Use Info Source: Partner | -0.786 | -0.854 | -0.719 |
| Use Info Source: TV | -0.768 | -0.812 | -0.723 |
| Use Info Source: Social Media | -0.799 | -0.843 | -0.755 |
| Use Info Source: Web COVID-19 Info Search: | -0.537 | -0.582 | -0.492 |
| Perceived Risk | -0.170 | -0.192 | -0.149 |
| Perceived Severity | -0.542 | -0.560 | -0.524 |
| Race: Interracial, Mixed Race, Or Other | -1.506 | -1.704 | -1.308 |
| Currently Married | 0.873 | 0.788 | 0.958 |
| Urban | 0.966 | 0.891 | 1.041 |
| Student/Unpaid Work | -2.942 | -3.082 | -2.803 |
| Essential Worker | 0.556 | 0.491 | 0.621 |
| Has Child(ren) | 0.628 | 0.506 | 0.750 |
| Income: 30,000 To Less Than 50,000 | -1.209 | -1.283 | -1.136 |
| Income: 75,000 To Less Than 100,000 | 0.561 | 0.523 | 0.598 |
| Republican | 0.422 | 0.371 | 0.473 |
| Region: Midwest | -0.564 | -0.621 | -0.507 |
| Region: West | 0.437 | 0.390 | 0.485 |
